# Recent Consanguinity and Outbred Autozygosity Are Associated With Increased Risk of Late-Onset Alzheimer’s Disease

**DOI:** 10.3389/fgene.2020.629373

**Published:** 2021-01-29

**Authors:** Valerio Napolioni, Marzia A. Scelsi, Raiyan R. Khan, Andre Altmann, Michael D. Greicius

**Affiliations:** ^1^Genomic and Molecular Epidemiology (GAME) Lab, School of Biosciences and Veterinary Medicine, University of Camerino, Camerino, Italy; ^2^Computational Biology in Imaging and Genetics (COMBINE) Lab, Centre for Medical Image Computing, Department of Medical Physics and Biomedical Engineering, University College London, London, United Kingdom; ^3^Department of Computer Science, Columbia University, New York, NY, United States; ^4^Functional Imaging in Neuropsychiatric Disorders (FIND) Lab, Department of Neurology and Neurological Sciences, Stanford University School of Medicine, Stanford, CA, United States

**Keywords:** Alzheimer disease, autozygosity, ethnic differences, directional dominance, inbreeding, recessive inheritance, runs of homozygosity (ROH), uniparental isodisomy

## Abstract

Prior work in late-onset Alzheimer’s disease (LOAD) has resulted in discrepant findings as to whether recent consanguinity and outbred autozygosity are associated with LOAD risk. In the current study, we tested the association between consanguinity and outbred autozygosity with LOAD in the largest such analysis to date, in which 20 LOAD GWAS datasets were retrieved through public databases. Our analyses were restricted to eight distinct ethnic groups: African–Caribbean, Ashkenazi–Jewish European, European–Caribbean, French–Canadian, Finnish European, North-Western European, South-Eastern European, and Yoruba African for a total of 21,492 unrelated subjects (11,196 LOAD and 10,296 controls). Recent consanguinity determination was performed using FSuite v1.0.3, according to subjects’ ancestral background. The level of autozygosity in the outbred population was assessed by calculating inbreeding estimates based on the proportion (F_ROH_) and the number (N_ROH_) of runs of homozygosity (ROHs). We analyzed all eight ethnic groups using a fixed-effect meta-analysis, which showed a significant association of recent consanguinity with LOAD (*N* = 21,481; OR = 1.262, *P* = 3.6 × 10^–4^), independently of *APOE*^∗^4 (*N* = 21,468, OR = 1.237, *P* = 0.002), and years of education (*N* = 9,257; OR = 1.274, *P* = 0.020). Autozygosity in the outbred population was also associated with an increased risk of LOAD, both for *F*_ROH_ (*N* = 20,237; OR = 1.204, *P* = 0.030) and *N*_ROH_ metrics (*N* = 20,237; OR = 1.019, *P* = 0.006), independently of *APOE*^∗^4 [(*F*_ROH_, *N* = 20,225; OR = 1.222, *P* = 0.029) (*N*_ROH_, *N* = 20,225; OR = 1.019, *P* = 0.007)]. By leveraging the Alzheimer’s Disease Sequencing Project (ADSP) whole-exome sequencing (WES) data, we determined that LOAD subjects do not show an enrichment of rare, risk-enhancing minor homozygote variants compared to the control population. A two-stage recessive GWAS using ADSP data from 201 consanguineous subjects in the discovery phase followed by validation in 10,469 subjects led to the identification of *RPH3AL* p.A303V (rs117190076) as a rare minor homozygote variant increasing the risk of LOAD [discovery: Genotype Relative Risk (GRR) = 46, *P* = 2.16 × 10^–6^; validation: GRR = 1.9, *P* = 8.0 × 10^–4^]. These results confirm that recent consanguinity and autozygosity in the outbred population increase risk for LOAD. Subsequent work, with increased samples sizes of consanguineous subjects, should accelerate the discovery of non-additive genetic effects in LOAD.

## Introduction

The impact of consanguinity on reproduction and Mendelian disorders is well known and documented (Bittles and Black, 2010). In contrast, very little has been published on the effects of consanguinity on late-onset diseases, even though inbreeding may have a prominent influence on late-onset traits (Rudan et al., 2003). Recessive inheritance of complex phenotypes can be linked to long [≥1-megabase (Mb)] runs of homozygosity (ROHs), which are indicative of recent consanguinity (Ghani et al., 2013). Levels of homozygosity vary by population owing to the evolutionary distance of different populations from the ancient migration events that led to elevated homozygosity (Pemberton et al., 2012; Kang et al., 2016).

Several studies have been carried out in late-onset Alzheimer’s Disease (LOAD) cohorts from different ethnicities, including Caribbean-Hispanics (Ghani et al., 2013), African Americans (Ghani et al., 2015), Wadi-Ara Arabs (Sherva et al., 2011), and Northern-Europeans (Nalls et al., 2009a; Sims et al., 2011) with the aim of determining the impact of ROHs on LOAD. The studies carried out in Caribbean-Hispanic (Ghani et al., 2013) and African American (Ghani et al., 2015) populations both demonstrated an association of long ROHs with LOAD, thus suggesting a link between recent consanguinity and LOAD. An association between consanguinity and LOAD was also demonstrated in a genealogical study of the Saguenay region in Québec (Vézina et al., 1999). Conversely, in the small ethnic isolate of Wadi-Ara Arabs (Sherva et al., 2011), the average degree of inbreeding was significantly higher in controls compared to cases. Moreover, the two studies carried out in Caucasians (Nalls et al., 2009a; Sims et al., 2011) showed discordant findings: the British–Irish study (Sims et al., 2011) displayed no association of number of ROHs with LOAD, while the mainly North-Western European cohort of neuropathologically verified subjects from the TGenII cohort (Nalls et al., 2009a) showed a suggestive increased number of ROHs in LOAD cases compared to controls. In sum, the results vary considerably by ancestral background, thus failing to provide a clear picture of the overall impact of homozygosity on LOAD risk.

In the present work we tested the association of consanguinity and autozygosity with LOAD by leveraging a large collection of publicly available GWAS data. To this aim, we determined the individual ancestry of subjects belonging to 20 independent GWAS datasets and pooled the consanguineous subjects according to their respective ethnic group. This step was followed by an association analysis between consanguinity in LOAD cases against older, cognitively healthy controls. We also tested the overall impact of genome-wide autozygosity in the outbred population. Finally, we leveraged Whole-Exome Sequencing (WES) data from the Alzheimer’s Disease Sequencing Project (ADSP) (Beecham et al., 2017) to test the global burden of rare minor homozygote variants in LOAD and to perform a two-stage recessive GWAS using 201 consanguineous subjects in the discovery phase followed by validation in 10,469 subjects.

## Materials and Methods

### Subjects

Twenty LOAD GWAS datasets were obtained from publicly available data repositories (Supplementary Table S1). The 20 datasets have been described in previous studies (Li et al., 2008; Filippini et al., 2009; Lee et al., 2011; Naj et al., 2011; Zhang et al., 2013; Clark et al., 2014; Proitsi et al., 2014; Saykin et al., 2015). Details of the participating studies and genotyping platforms used are provided in Supplementary Table S1.

The Alzheimer’s Disease Neuroimaging Initiative (ADNI) (Saykin et al., 2015) was launched in 2003 as a public-private partnership, led by Principal Investigator Michael W. Weiner, MD. The primary goal of ADNI has been to test whether serial magnetic resonance imaging, positron emission tomography, other biological markers, and clinical and neuropsychological assessment can be combined to measure the progression of mild cognitive impairment and early Alzheimer’s disease.

Whole-exome sequencing from the discovery phase of the Alzheimer’s Disease Sequencing Project (ADSP) (Beecham et al., 2017) was obtained through the National Institute on Aging Genetics of Alzheimer’s Disease Data Storage Site (NIAGADS) and it includes 5,096 Alzheimer’s Disease (AD) cases and 4,965 controls, with an additional enriched sample set comprised of 853 AD cases from multiple affected families and 171 Hispanic controls.

This was a re-analysis of de-identified data available from shared data repositories. The study protocol was granted an exemption by the Stanford Institutional Review Board because the analyses were carried out on “de-identified, off-the-shelf” data.

### Inclusion Criteria, Quality Control (QC) Pipeline, Ancestry Determination and Imputation

**FIGURE 1:**
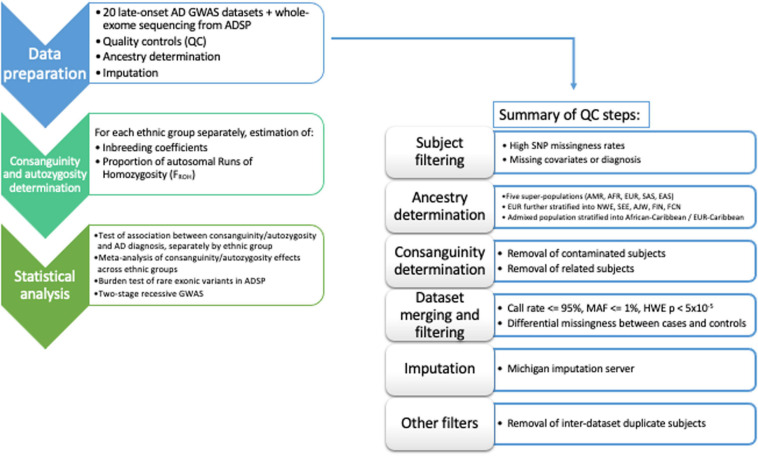
Flow-chart of the workflow adopted in the data QC/harmonization/ancestry-determination processing of the full dataset.

The entire dataset includes 34,111 participants. Analyses were performed using PLINK 1.9 (Chang et al., 2015). A comprehensive flowchart of the data QC/harmonization/ancestry-determination steps applied to the full dataset is reported as Figure 1.

Subjects with autosome missingness (≥5%) and/or X-chromosome missingness (≥5%) within the same dataset, age below 60 years, age information missing, or phenotype inconsistency [missing phenotype, diagnosis of mild cognitive impairment or other neurodegenerative phenotype] were excluded from the analysis (Supplementary Table S2).

Individual ancestry was determined using SNPweights v.2.1 (Chen et al., 2013) using reference populations from the 1000 Genomes Project (1KGP) (1000 Genomes Project Consortium, Auton et al., 2015). By applying an ancestry percentage cut-off ≥ 80%, the samples were stratified into the five super populations, South-Asians (SAS), East-Asians (EAS), Americans (AMR), Africans (AFR), and Europeans (EUR) (Supplementary Table S2). Since most of the samples belonged to the European population, we also determined their ancestry percentage according to four major ethnicities, North-Western, South-Eastern, Ashkenazi-Jewish, and Finnish Europeans, using reference populations available both from SNPweights v.2.1 (Chen et al., 2013) and 1KGP (1000 Genomes Project Consortium, Auton et al., 2015). European subjects were stratified into the above-mentioned ethnicities when their ancestry percentage was attributable with an ancestry percentage cut-off ≥ 50% (Supplementary Table S3).

We assigned French–Canadian (FCN) ancestry to subjects included in GenADA GWAS (Li et al., 2008) when they both reported Canada as country of origin for all the grandparents and French as their first spoken language.

Most subjects belonging to the Columbia University Study of Caribbean Hispanics with Familial and Sporadic Late Onset Alzheimer’s disease (CIDR) (Lee et al., 2011) had admixed ancestry (Supplementary Table S2); therefore, we stratified the subjects into three groups according to their prevalent ancestral background. This stratification allowed the definition of one dataset composed of African–Caribbean (African ancestry ≥ 50%), one dataset composed of European–Caribbean (European ancestry ≥ 50%) and one dataset including highly admixed subjects (ancestry percentage less than 50% attributable to a unique super-population from 1KGP, 1000 Genomes Project Consortium, Auton et al., 2015). Only the African–Caribbean and the European–Caribbean datasets were considered for the analyses. Subjects with genetic ancestry estimates discordant from self-reported ancestry were excluded from the analyses.

Next, datasets were tested for presence of consanguineous subjects using FSuite v.1.0.3 (Gazal et al., 2014). Consanguineous female subjects were flagged to avoid their exclusion because of apparent sex-inconsistency (e.g., due to increased homozygosity at X-chromosome SNPs). Subjects showing sex-inconsistency were excluded along with the possibly contaminated samples {heterozygosity *F* ≤ −0.03; more than 25 related [(identity-by-descent (IBD) ≥ 0.0625, equivalent to 3rd degree relative] within the same dataset}.

With the aim of maximizing the efficiency of quality control procedures and harmonizing the GWAS results after imputation, we collapsed the genotyping data from the 20 GWAS (when needed), according to sample ethnicity and the number of SNPs shared across the SNP-array platforms. Thus, we defined five groups, reported in Supplementary Table S4, where the subjects from different GWAS were collapsed and further QCed to remove the SNPs with a call rate ≤ 95%; Minor Allele Frequency (MAF) ≤ 1%; SNPs with MAF deviating more than 10% from the MAF reported in 1KGP for the relative population; SNPs with differential missingness between cases and controls (*P* < 0.05); SNPs deviating from Hardy–Weinberg Equilibrium (HWE) in controls (*P* < 5 × 10^–5^); tri-allelic SNPs; and SNPs where the alleles are mismatched compared to the 1KGP reference sequence. A/T and C/G SNPs were removed prior to imputation.

All the datasets were phased and imputed using the Michigan Imputation Server (Das et al., 2016), considering the Haplotype Reference Consortium r1.1. 2016 European panel (McCarthy et al., 2016) for Europeans, 1KGP Phase 3 African panel (1000 Genomes Project Consortium, Auton et al., 2015) for African Indianapolis-Ibadan and the Consortium on Asthma among African-ancestry Populations in the Americas (CAAPA) (Mathias et al., 2016) for admixed Caribbean. After imputation, SNPs with a *r*^2^ quality score ≤ 0.7 and MAF ≤ 0.01 were excluded. Results of the imputation process are provided in Supplementary Table S5 and Supplementary Figure S1.

For the statistical analyses, inter-dataset duplicates (IBD ≥ 0.95) were removed from the dataset having the lowest SNP coverage, while, in case of relatedness (IBD ≥ 0.0625) the affected or older subject were kept, independently of SNP coverage (Supplementary Tables S6–S13).

### Consanguinity Determination

Consanguinity determination was performed using FSuite v1.0.3 (Gazal et al., 2014), both at the pre- and post-imputation stage on QCed SNPs, according to each subjects’ ancestral background. Results were concordant in 98% of the subjects analyzed. Discordant subjects were kept in the subsequent analyses as outbred, since their ROHs may be somatic Copy Number Variations or linked to a specific ancestral background (e.g., ethnic minorities – Acadians, Sardinians, etc.) not captured by our ancestry-determination pipeline.

Subjects showing a homozygous region over 10 Mb on only one chromosome were considered carriers of putative uniparental isodisomy (UPD), according to the homozygosity cut-off previously reported (Papenhausen et al., 2011). UPD carriers were excluded from the association testing of consanguinity with LOAD since they represent subjects affected by chromosomal alterations, not the result of consanguineous unions.

### ROH Calling and Burden Analysis

Runs of homozygosity (≥1 Mb) were determined for each ethnic group separately using PLINK1.9 (Chang et al., 2015) and according to the guidelines recently reported (Keller et al., 2012). GWAS datasets were pruned for strong LD (MAF ≥ 0.01, *r*^2^ ≤ 0.1) and ROHs were defined as being ≥ 65 consecutive homozygous SNPs with no heterozygote calls allowed and a density greater than 1 SNP per 200 kb.

For each subject, we summed the total length of all their ROHs in the autosome and divided by the total SNP-mappable autosomal distance (2.77 × 10^9^ bases) to derive *F*_ROH_, the proportion (between 0 and 1) of the autosome in ROHs, as previously described (Chang et al., 2015). *F*_ROH_ was used as the predictor of case-control status in ROH burden analyses.

### Population Genetics, WES Variant Annotation and Statistical Analyses

Inter-population structure was examined using ADMIXTURE (Alexander et al., 2009). Intra-population structure for each ancestral group was determined using principal components (PCs) obtained from EIGENSOFT v.6.1 (Price et al., 2006) using pruned (*r*^2^ ≤ 0.1), directly genotyped, SNPs.

The association of consanguinity/autozygosity with LOAD was carried out using three different logistic regression models:

(a) MODEL1: adjusting for each subject’s age at LOAD onset (when not available, we used the subject’s age at last visit or death), sex, the first three PC eigenvalues from population structure and GWAS imputation group (only for Ashkenazi-Jewish Europeans, Finnish Europeans, North-Western Europeans and South-Eastern Europeans, since those subjects were genotyped on multiple platforms);
(b) MODEL2: including *APOE*^∗^4 dose to the list of covariates used in MODEL1;
(c) MODEL3: including “years of education” (EDU) to the list of covariates used in MODEL2.

Since EDU is available only for 43.1% of the full dataset (9,260 out of 21,492 subjects), we fitted MODEL1 and MODEL2 to this restricted subset of subjects as well, to allow an appropriate comparison with MODEL3 of the effect estimates. The association of EDU with *F*_ROH_ was tested by linear regression, considering MODEL2 and adjusting for diagnosis status. The analyses were carried out for each ethnic group separately and combined using a fixed-effect meta-analysis implemented in GWAMA (Mägi and Morris, 2010).

GWAS participants were mapped to ADSP WES participants through IBD estimation using ∼30K common, overlapping SNPs (MAF > 0.01) between the two datasets. We considered matching samples as those with a pair-wise coefficient of relatedness above 99%. The burden of rare recessive variants was tested using a general linear model, adjusting for sex, age, *APOE*^∗^4 dose and ethnicity. ADSP WES data were annotated using Variant Effect Predictor (VEP) (McLaren et al., 2016). Recessive variants having a CADD (Rentzsch et al., 2019) score ≥ 15 and a MAF < 10% were considered when testing the global burden of rare recessive variants in LOAD and in a two-stage GWAS by applying the Recessive-Allele Frequency Test (RAFT) statistic (Lim et al., 2014).

Statistical significance was set at *P* < 0.05 for all the association testing, while for the two-stage GWAS, we applied Bonferroni’s correction according to the number of recessive variants tested in the discovery [*P* < 1.8 × 10^–5^ (0.05/2767)] and replication [*P* < 0.007 (0.05/7)] phases.

## Results

### Ancestry Determination and Ethnic-Specific Differences

**FIGURE 2:**
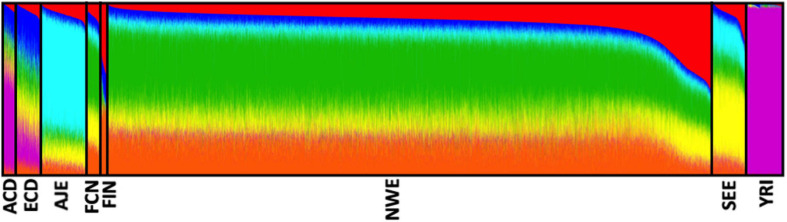
Analysis of the genetic structure confirmed the effectiveness of our ancestry-determination pipeline. Ancestry proportions of the 21,492 unrelated subjects studied (from the eight determined ethnic groups) as revealed by the ADMIXTURE software (Alexander et al., 2009), using pruned (MAF ≥ 0.01, *r*^2^ ≤ 0.1) SNPs at *K* = 6. Each color represents a different ancestral component, and each ancestry is a mixture of different components. ACD, African–Caribbean from Dominican Republic; AJE, Ashkenazi-Jewish Europeans; ECD, European–Caribbean from Dominican Republic; FCN, French–Canadians; FIN, Finnish Europeans; NWE, North-Western Europeans; SEE, South-Eastern Europeans; YRI, African Yoruba.

After quality control procedures, our analyses were restricted to eight distinct ethnic groups, namely African–Caribbeans from Dominican Republic (ACD, *N* = 398), Ashkenazi-Jewish Europeans (AJE, *N* = 1,229), European–Caribbeans from Dominican Republic (ECD, *N* = 671), French–Canadians (FCN, *N* = 376), Finnish Europeans (FIN, *N* = 219), North-Western Europeans (NWE, *N* = 16,496), South-Eastern Europeans (SEE, *N* = 1,083), and African-Yoruba (YRI, *N* = 1,020), for a total of 21,492 unrelated subjects (11,196 LOAD, 10,296 controls). The analysis of genetic structure, applied to the whole dataset, confirmed the effectiveness of ancestry determination cut-offs (Figure 2).

**FIGURE 3:**
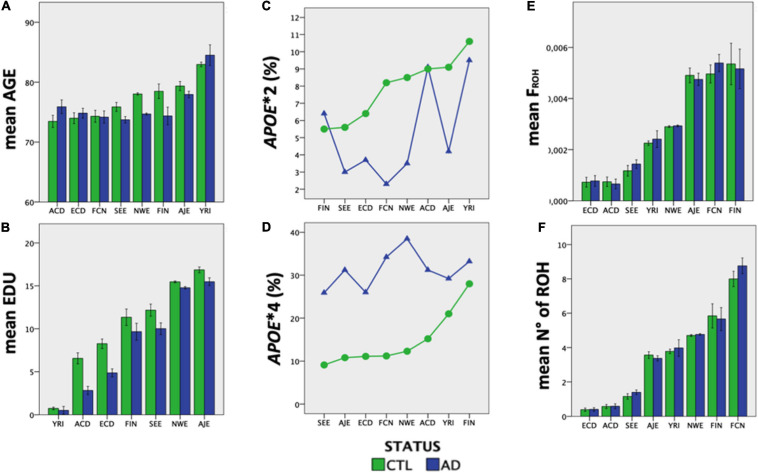
The eight ethnic groups analyzed showed ethnic-specific differences in Alzheimer’s-relevant risk factors. **(A)** mean age, **(B)** EDU, **(C)** *APOE^∗^2* frequency, **(D)** *APOE^∗^4* frequency, **(E)** inbreeding coefficient, and **(F)** number of ROHs (>1 Mb) differ significantly across the eight ethnic groups analyzed (*P* < 0.00001).

Ethnic groups showed significant differences (*P* < 0.00001) in mean age, EDU and *APOE* allele frequency, independently of diagnostic status (Figure 3). Both consanguinity rates and consanguinity degree differed significantly (*P* < 0.00001) across the eight ethnic groups analyzed, with percentages of consanguineous subjects ranging from 1.2% in YRI to 31.7% in ECD (Table 1). As expected, consanguineous subjects displayed higher *F*_ROH_ estimates (0.024 ± 0.025 vs. 0.003 ± 0.002) and more ROH (*N*_ROH_, 9.4 ± 6.3 vs. 4.4 ± 2.5) compared to outbred subjects (*P* < 0.00001, Table 2). When considering exclusively the outbred population, both *F*_ROH_ estimates and *N*_ROH_ significantly differed across the eight ethnic groups analyzed (*P* < 0.00001, Figure 3), with ACD and ECD showing the lowest *F*_ROH_ (0.0007 ± 0.0012) and *N*_ROH_ (0.4 ± 0.8), respectively. Conversely, the highest *F*_ROH_ and *N*_ROH_ were found in FIN (0.0053 ± 0.0038) and FCN (8.4 ± 2.9), respectively (Figure 3).

**TABLE 1:** Consanguinity rates differ across the eight ethnic groups analyzed.

**Ethnicity**	**Status**	***N***	**INBRED *N* (%)**	**1C N (INBRED-%)**	**2C N (INBRED-%)**	**2 × 1C N (INBRED-%)**	**AV N (INBRED-%)**
ACD	Total	398	94 (23.6)	13 (13.8)	80 (85.1)	—	1 (1.1)
	LOAD	170	47 (27.7)	10 (21.3)	37 (81.2)	—	—
	Controls	228	47 (20.6)	3 (6.4)	43 (91.5)	—	1 (2.1)
AJE	Total	1229	141 (11.5)	34 (24.1)	107 (75.8)	—	1 (0.1)
	LOAD	754	80 (10.6)	21 (26.3)	59 (73.8)	—	—
	Controls	475	61 (12.8)	13 (21.3)	48 (78.7)	—	1 (1.6)
ECD	Total	671	213 (31.7)	49 (23.0)	158 (74.2)	5 (2.3)	1 (0.5)
	LOAD	337	117 (34.7)	32 (27.4)	80 (68.4)	4 (3.4)	1 (0.8)
	Controls	334	96 (28.7)	17 (17.7)	78 (81.3)	1 (1.0)	—
FCN	Total	376	53 (14.1)	3 (5.7)	50 (94.3)	—	—
	LOAD	193	29 (15.0)	1 (3.4)	28 (96.6)	—	—
	Controls	183	24 (13.1)	2 (8.3)	22 (91.7)	—	—
FIN	Total	219	31 (14.2)	—	31 (100.0)	—	
	LOAD	110	18 (16.4)	—	18 (100.0)	—	—
	Controls	109	13 (11.9)	—	13 (100.0)	—	—
NWE	Total	16,496	500 (3.0)	55 (11.0)	439 (87.8)	6 (1.2)	—
	LOAD	8,870	313 (3.5)	37 (11.8)	270 (86.3)	6 (1.9)	—
	Controls	7,626	187 (2.5)	18 (9.6)	169 (90.4)	—	—
SEE	Total	1,083	184 (17.0)	13 (7.1)	171 (92.9)	—	—
	LOAD	678	116 (17.1)	10 (8.6)	106 (91.4)	—	—
	Controls	405	68 (16.8)	3 (4.4)	65 (95.6)	—	—
YRI	Total	1,020	12 (1.2)	—	12 (100.0)	—	—
	LOAD	84	1 (1.2)	—	1 (100.0)	—	—
	Controls	936	11 (1.2)	—	11 (100.0)	—	—

*1C, first-cousins offspring; 2C, second-cousins offspring; 2 × 1C, double-first cousins offspring; AV, avuncular offspring. ACD, African–Caribbean from Dominican Republic; AJE, Ashkenazi-Jewish Europeans; ECD, European–Caribbean from Dominican Republic; FCN, French–Canadians; FIN, Finnish Europeans; NWE, North-Western Europeans; SEE, South-Eastern Europeans; YRI, African Yoruba.*

**TABLE 2:** Genome-wide autozygosity determined through *F*_ROH_ estimates and number of ROHs across the eight ethnic groups according to case-control and consanguinity status.

**Ethnicity**	**Status**	**Outbred *F*_ROH_ (Mean ± SD)**	**Inbred *F*_ROH_ (Mean ± SD)**	**Outbred ROH N (Mean ± SD)**	**Inbred ROH N (Mean ± SD)**
ACD	Total	0.0007 ± 0.0012	0.0251 ± 0.0328	0.6 ± 0.8	6.6 ± 5.5
	LOAD	0.0006 ± 0.0011	0.0280 ± 0.0233	0.6 ± 0.8	7.8 ± 5.6
	Controls	0.0007 ± 0.0012	0.0223 ± 0.0402	0.6 ± 0.8	5.4 ± 5.1
AJE	Total	0.0048 ± 0.0031	0.0286 ± 0.0255	3.4 ± 2.0	9.3 ± 5.4
	LOAD	0.0048 ± 0.0032	0.0287 ± 0.0233	3.4 ± 2.0	9.0 ± 5.3
	Controls	0.0049 ± 0.0030	0.0285 ± 0.0284	3.6 ± 2.0	9.6 ± 5.7
ECD	Total	0.0008 ± 0.0015	0.0275 ± 0.0303	0.4 ± 0.8	6.9 ± 5.8
	LOAD	0.0007 ± 0.0016	0.0313 ± 0.0349	0.4 ± 0.8	7.9 ± 6.6
	Controls	0.0007 ± 0.0015	0.0229 ± 0.0228	0.4 ± 0.8	5.8 ± 4.5
FCN	Total	0.0052 ± 0.0022	0.0227 ± 0.0154	8.4 ± 2.9	13.9 ± 5.1
	LOAD	0.0054 ± 0.0022	0.0238 ± 0.0165	8.8 ± 3.0	14.6 ± 5.3
	Controls	0.0050 ± 0.0023	0.0215 ± 0.0141	8.0 ± 2.9	13.0 ± 4.9
FIN	Total	0.0053 ± 0.0038	0.0156 ± 0.0067	5.8 ± 3.3	10.8 ± 3.3
	LOAD	0.0052 ± 0.0036	0.0162 ± 0.0077	5.7 ± 3.2	11.1 ± 3.5
	Controls	0.0053 ± 0.0040	0.0148 ± 0.0054	5.8 ± 3.5	10.4 ± 3.1
NWE	Total	0.0029 ± 0.0016	0.0233 ± 0.0233	4.7 ± 2.3	11.3 ± 6.7
	LOAD	0.0029 ± 0.0016	0.0242 ± 0.0264	4.8 ± 2.3	11.4 ± 7.2
	Controls	0.0029 ± 0.0016	0.0219 ± 0.0173	4.7 ± 2.3	11.2 ± 5.9
SEE	Total	0.0013 ± 0.0020	0.0198 ± 0.0182	1.3 ± 1.6	7.9 ± 5.2
	LOAD	0.0014 ± 0.0020	0.0200 ± 0.0202	1.4 ± 1.6	7.7 ± 5.4
	Controls	0.0012 ± 0.0019	0.0193 ± 0.0143	1.2 ± 1.5	8.2 ± 4.8
YRI	Total	0.0022 ± 0.0014	0.0111 ± 0.0059	3.8 ± 2.0	6.6 ± 1.8
	LOAD	0.0024 ± 0.0015	0.0078	4.0 ± 2.2	7
	Controls	0.0023 ± 0.0014	0.0113 ± 0.0060	3.8 ± 2.0	6.5 ± 1.9

*ACD, African–Caribbean from Dominican Republic; AJE, Ashkenazi-Jewish Europeans; ECD, European–Caribbean from Dominican Republic; FCN, French–Canadians; FIN, Finnish Europeans; NWE, North-Western Europeans; SEE, South-Eastern Europeans; YRI, African Yoruba.*

Consanguineous subjects in ACD, ECD, FIN, and NWE groups showed a statistically significant (ACD, ECD, NWE), or nearly significant (FIN), lower education level compared to the relative outbred population (on average, 1.4 fewer EDU, Supplementary Tables S6–S13). Conversely, YRI consanguineous subjects showed higher education levels compared to the relative outbred population (Supplementary Table S13). No association was detected between *F*_ROH_ and EDU in the outbred sample (*N* = 8,648, β = −0.059, *SE* = 0.196, *P* = 0.760, heterogeneity-*Q* = 2.1 × 10^–7^). However, a highly significant heterogeneity was found across seven ethnic groups, with ACD (β = −3.765, *P* = 0.077), ECD (β = −2.688, *P* = 0.059), and FIN (β = −4.959, *P* < 0.00001) showing a significant, or nearly significant, negative correlation between *F*_ROH_ and EDU, while YRI displayed an opposite correlation (β = 0.870, *P* = 0.079).

No significant differences were found for mean age between inbred and outbred groups. Consanguinity rates reported for the eight analyzed ethnic groups largely fall within the ranges reported in the literature (Goldschmidt et al., 1960; Gazal et al., 2015; Vardarajan et al., 2015).

The distribution of *APOE* genotypes and alleles did not show deviations from HWE (Supplementary Table S14). In addition, *APOE* genotypes and allele counts differed significantly between outbred and consanguineous subjects only for AJE controls, FCN cases and FIN controls (Supplementary Table S14).

### Consanguinity Is Associated With an Increased Risk of LOAD

Consanguinity was significantly associated with LOAD (*N* = 21,481, OR = 1.262, *P* = 3.6 × 10^–4^), independently of *APOE*^∗^4 (*N* = 21,468, OR = 1.237, *P* = 0.002) and EDU (*N* = 9,257, OR = 1.274, *P* = 0.020) (Table 3).

**TABLE 3:** Consanguinity increases the risk of LOAD.

**Full dataset**														
**MODEL1**	**OR**	**95% CI**	**P**	**q_p-value**	**I2**	**N**	**ACD**	**AJE**	**ECD**	**FCN**	**FIN**	**NWE**	**SEE**	**YRI**

Consanguinity	1.262	1.111–1.435	*3.58E-04*	0.200	0.285	21,481	+	–	+	+	+	+	+	+
Close consanguinity (1C+2 × 1C+AV)	1.713	1.226–2.394	*0.002*	0.385	0.049	19,227	+	–	+	–	NA	+	+	NA
Distant consanguinity (2C)	1.207	1.054–1.383	*0.007*	0.295	0.171	21,284	+	–	–	+	+	+	+	+
**MODEL2**														
Consanguinity	1.237	1.081–1.417	*0.002*	0.864	0.000	21,468	+	+	+	+	+	+	+	+
Close consanguinity (1C+2 × 1C+AV)	1.798	1.267–2.552	*0.001*	0.681	0.000	19,212	+	+	+	+	NA	+	+	NA
Distant consanguinity (2C)	1.172	1.015–1.354	*0.031*	0.883	0.000	21,271	+	+	–	+	+	+	+	+

**EDU Subset**														

**MODEL1**														
Consanguinity	1.423	1.180–1.716	*2.21E-04*	0.549	0.000	9,260	+	+	+	NA	+	+	+	+
Close consanguinity (1C + 2 × 1C + AV)	2.201	1.401–3.455	*6.16E-04*	0.487	0.000	7,619	+	+	+	NA	NA	+	+	NA
Distant consanguinity (2C)	1.312	1.074–1.603	*0.008*	0.530	0.000	9,156	+	+	–	NA	+	+	+	+
**MODEL2**														
Consanguinity	1.418	1.165-1.725	*5.04E-04*	0.792	0.000	9,252	+	+	+	NA	+	+	+	+
Close consanguinity (1C + 2 × 1C + AV)	2.417	1.516–3.854	*2.11E-04*	0.717	0.000	7,611	+	+	+	NA	NA	+	+	NA
Distant consanguinity (2C)	1.287	1.042–1.588	*0.019*	0.729	0.000	9,147	+	+	–	NA	+	+	+	+
**MODEL3**														
Consanguinity	1.274	1.038–1.562	*0.020*	0.483	0.000	9,252	+	+	–	NA	+	+	+	+
Close consanguinity (1C + 2 × 1C + AV)	2.014	1.240–3.271	*0.005*	0.612	0.000	7,611	+	+	+	NA	NA	+	+	NA
Distant consanguinity (2C)	1.170	0.941–1.455	0.158	0.459	0.000	9,147	+	+	–	NA	+	+	+	+

*ACD, African–Caribbean from Dominican Republic; AJE, Ashkenazi-Jewish Europeans; ECD, European–Caribbean from Dominican Republic; FCN, French–Canadians, FIN, Finnish Europeans; NWE, North-Western Europeans; SEE, South-Eastern Europeans; YRI, African Yoruba. 1C, first-cousins offspring; 2C, second-cousins offspring; 2 × 1C, double-first cousins offspring; AV, avuncular offspring; q p-value, Cochran’s heterogeneity statistic’s *p*-value; i2, heterogeneity index; ±, summary of effect directions; NA, not available. Values reported in italics are statistically significant.*

When testing the association of degree of consanguinity with LOAD by separating close (first cousin/double-first cousin/avuncular offspring) from distant (second cousin offspring) consanguinity, it became clear that the association was driven by close consanguinity (close: *N* = 19,227, OR = 1.713, *P* = 0.002; distant: *N* = 21,284, OR = 1.207, *P* = 0.007, Table 3). The association reported for close and distant consanguinity was independent of *APOE*^∗^4 and EDU (Table 3). When considering the analyses carried out in the smaller EDU subset, the inclusion of *APOE^∗^4* does not reduce statistical estimates of the associations (Table 3). Conversely, the inclusion of EDU as a variable slightly decreases all the associations reported in MODEL3 compared to MODEL2, such that the association of distant consanguinity with LOAD in MODEL3 trends in the same direction but is no longer statistically significant (OR = 1.170, *P* = 0.158, Table 3).

### Autozygosity in the Outbred Population Is Associated With an Increased Risk of LOAD

Table 4 reports the results obtained from the meta-analysis of the association of genome-wide autozygosity determined both by *F*_ROH_ estimates and by *N*_ROH_ across the eight ethnic groups. When considering the full dataset, both *F*_ROH_ and number of ROHs are significantly associated with LOAD, independently of *APOE*^∗^4 (Table 4). However, when testing the association of *F*_ROH_ and the number of ROHs with LOAD in the subset with information on EDU, the meta-analysis results are not statistically significant for Table 4, likely reflecting a lack of power rather than an effect of education given that MODELS 1 and 2 are also no longer significant with these sample sizes.

**TABLE 4:** Autozygosity in the outbred population increases the risk of LOAD.

**Full dataset**														
**MODEL1**	**OR**	**95% CI**	***P***	**q_p-value**	**i2**	***N***	**ACD**	**AJE**	**ECD**	**FCN**	**FIN**	**NWE**	**SEE**	**YRI**

*F*_ROH_	1.204	1.018–1.424	*0.030*	0.319	0.142	20,237	–	–	–	+	–	+	+	+
*N*_ROH_	1.019	1.005–1.034	*0.006*	0.220	0.261	20,237	+	–	–	+	–	+	+	+
**MODEL2**														
*F*_ROH_	1.222	1.021–1.462	*0.029*	0.245	0.232	20,225	–	–	+	+	–	+	+	+
*N*_ROH_	1.019	1.005–1.034	*0.007*	0.241	0.237	20,225	–	–	+	+	–	+	+	+

**EDU subset**														

**MODEL1**														
*F*_ROH_	1.141	0.871–1.494	0.340	0.821	0.000	8,655	–	+	–	NA	–	+	+	+
*N*_ROH_	1.009	0.988–1.031	0.413	0.683	0.049	8,655	+	+	–	NA	–	+	+	+
**MODEL2**														
*F*_ROH_	1.180	0.887–1.570	0.256	0.712	0.000	8,647	–	+	+	NA	–	+	+	+
*N*_ROH_	1.011	0.988–1.034	0.348	0.561	0.000	8,647	–	+	+	NA	–	+	+	+
**MODEL3**														
*F*_ROH_	1.123	0.839–1.503	0.435	0.392	0.046	8,647	–	+	–	NA	–	+	+	+
*N*_ROH_	1.011	0.989–1.034	0.334	0.258	0.224	8,647	–	+	–	NA	–	+	+	+

*ACD, African–Caribbean from Dominican Republic; AJE, Ashkenazi-Jewish Europeans; ECD, European–Caribbean from Dominican Republic; FCN, French–Canadians; FIN, Finnish Europeans; NWE, North-Western Europeans; SEE, South-Eastern Europeans; YRI, African Yoruba. N_ROH_, number of ROHs; Q *p*-value, Cochran’s heterogeneity statistic’s *p*-value; i2, heterogeneity index; ±, summary of effect directions; NA, not available. Values reported in italics are statistically significant.*

### LOAD Genome Is Not Enriched in Rare Recessive Damaging Variants

Given the consistent association of LOAD with consanguinity and autozygosity, we leveraged ADSP WES data to establish whether LOAD subjects showed an enrichment of damaging recessive variants compared to the control population. After merging GWAS imputed data from NWE, SEE, AJE, FIN, and FCN groups with ADSP WES data, we determined that 4,969 subjects were overlapping between the two datasets (AJE = 287; FIN = 47; NWE = 4,424; SEE = 211).

Previous studies have shown that long ROHs are enriched for damaging homozygous variants, with the majority having a MAF ≤ 5% (Pemberton et al., 2012; Pemberton and Szpiech, 2018). Thus, we determined the number of rare, deleterious minor homozygote variants (RMHV) for each GWAS subject that was also whole-exome sequenced through ADSP. The four ethnic groups differed significantly in the average individual number of RMHV in their respective outbred population (*N* = 4,753, *P* = 0.0002), independently of diagnostic status (AJE: 18.97 ± 0.31; FIN:16.65 ± 0.74; NWE: 14.88 ± 0.08; SEE: 18.16 ± 0.38). As expected, consanguineous subjects displayed a significantly higher average individual number of RMHV compared to the outbred population, independently of their ethnicity and diagnostic status (23.31 ± 0.37 vs. 15.26 ± 0.08, *P* < 0.00001). Notably, the average RMHV in 12 subjects carrying a putative UPD was lower than the one reported for the outbred group, and significantly different compared to distant or close consanguineous subjects (UPD:14.46 ± 1.43; outbred:15.26 ± 0.07; distant consanguinity:20.23 ± 0.39; close consanguinity:42.14 ± 1.43).

When testing the burden of RMHV in LOAD vs. controls, no significant association was detected in the outbred (LOAD:15.15 ± 0.10; Controls:15.35 ± 1.14; *P* = 0.303) or consanguineous (LOAD:23.97 ± 0.99; Controls:24.48 ± 1.67; *P* = 0.805) group.

### Identification of *RPH3AL* p.A303V (rs117190076) as RMHV Associated With LOAD

Despite the lack of association between the burden of RMHV and LOAD, we decided to leverage WES data to perform a two-stage recessive-GWAS using the 201 consanguineous subjects identified in ADSP as discovery phase, followed by validation in the remaining 10,469 ADSP subjects. To this aim, we applied the RAFT statistic (Lim et al., 2014) to the 2,767 RMHV detected in the discovery cohort composed exclusively of consanguineous subjects. Seven RMHV yielded a Bonferroni’s corrected statistically significant *P* < 1.8 × 10^–5^ (Table 5). When applying the RAFT statistic to the seven variants in the validation group, only the *RPH3AL* missense variant (rs117190076, NP_001177340 p.A303V), successfully replicated (Genotype Relative Risk = 1.9, *P* = 8.0 × 10^–4^). However, we could not validate one of the seven variants passing the statistical threshold in the discovery phase (*SCAPER* on chr15q24.3, rs200719909, NP_001339938 p.A280V), because no minor homozygote was detected in the replication/validation phase conducted on outbred subjects (Table 5). Remarkably, 523 out of 2,767 RMHV tested in the discovery group (18.9%) did not have a minor homozygote counterpart in the validation group (Supplementary Table S15). Although those variants did not pass the statistical threshold, set up by applying the Bonferroni’s correction in the discovery phase on outbred subjects, they may still have functional/causal role in LOAD.

**TABLE 5:** Identification of *RPH3AL* rs117190076 as rare, deleterious minor homozygote variant associated with LOAD.

					**Discovery Phase**								**Validation Phase**							
						
**Gene**	**rsID**	**GnomAD_GlobalMAF**	**Consequence**	**CADD**	**AA_LOAD**	**Aa_LOAD**	**aa_LOAD**	**AA_CONTROLS**	**Aa_CONTROLS**	**aa_CONTROLS**	**GRR**	**P-value**	**AA_LOAD**	**Aa_LOAD**	**aa_LOAD**	**AA_CONTROLS**	**Aa_CONTROLS**	**aa_CONTROLS**	**GRR**	**P-value**
*CLEC1B*	rs2273987	0.0802	NP_ 057593.3, p.Ser28Phe	25	117	21	4	58	1	0	404	2.66 × 10^–10^	4,773	895	51	3,998	710	38	1.100	0.451
*KNDC1*	rs11101618	0.0220	NP_ 689856.6, p.Ala128Ser	24	134	3	3	58	1	0	305	1.05 × 10^–7^	5,385	287	4	4,413	281	7	1.000	1.000
*SCAPER*	rs200719909	0.0004	NP_ 001339938.1, p.Ala280Val	15	140	0	2	59	0	0	2309	2.09 × 10^–7^	5,714	4	0	4,736	4	0	—	—
*IGSF22*	rs78892734	0.0375	NP_ 775859.3, p.Ala1008Thr	21	125	4	2	48	0	0	1987	2.85 × 10^–7^	4,838	306	17	4,036	232	10	1.400	0.181
*C16orf74*	rs141454251	0.0092	NP_ 996850.1, p.Gln12His	23	118	0	2	42	0	0	1779	3.59 × 10^–7^	4,982	100	1	3,911	64	1	1.000	1.000
*SGSH*	rs9894254	0.0528	NP_ 000190.1, p.Val361Ile	23	113	9	3	37	1	0	142	1.12 × 10^–6^	4,695	333	13	3,627	278	9	1.100	0.689
*RPH3AL*	rs117190076	0.0534	NP_ 001177340.1, p.Ala303Val	18	120	14	4	55	3	0	45	2.16 × 10^–6^	4,988	603	36	4,138	477	16	1.900	0.001

*AA, major homozygote; Aa, heterozygote; aa, minor homozygote; GRR, Genotype Relative Risk.*

### Putative UniParental Disomy Does Not Associate With LOAD

**FIGURE 4:**
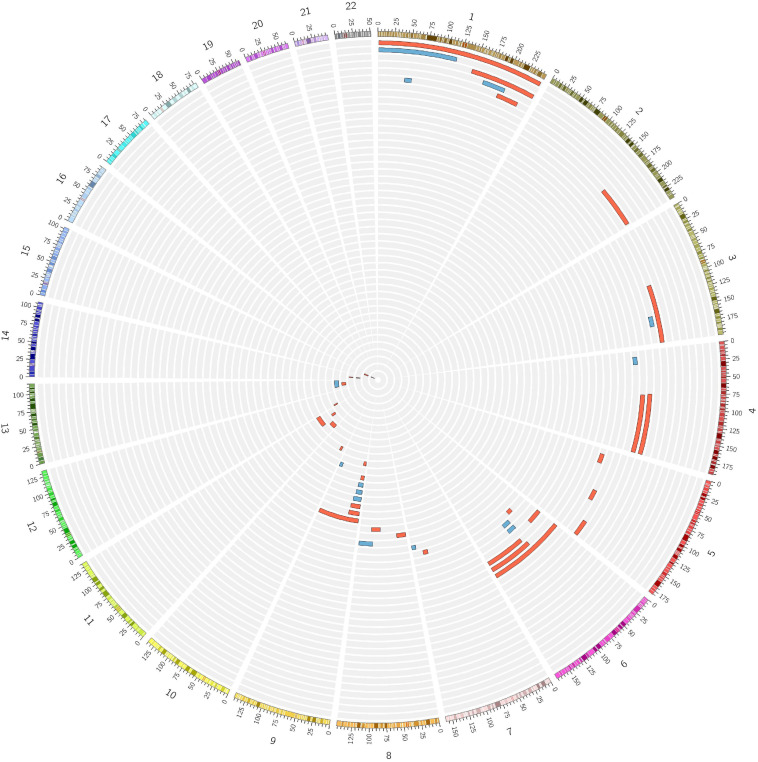
Numerous cases of putative isodisomy misidentified as consanguineous subjects. Fifty-six subjects identified as consanguineous by FSuite v1.0.3 (Gazal et al., 2014) showed a single homozygous region over 10 Mb on just one chromosome, the homozygosity cut-off previously reported to define the presence of putative uniparental isodisomy (UPD). Two female subjects showing a putative UPD on X-chromosome are not shown. Red = LOAD; Blue = Control.

During the inbreeding determination process, 56 subjects (6 ACD, 42 NWE, 8 SEE) were found to be potential cases of UPD (Figure 4). The presence of UPD did not show a significant association with an increased risk for LOAD in a logistic regression testing the presence of putative isodisomy compared to the rest of ACD, NWE, and SEE outbred populations (OR = 1.561, *P* = 0.158). The origin of UPD in our subjects is unknown due to the lack of genotype data from their parents. Nine putative UPD subjects had first-degree relatives genotyped and in these nine cases none of the first-degree relatives showed any evidence of consanguinity or shared long ROHs, suggesting the presence of true isodisomy. Moreover, one of the 12 UPD subjects had ADSP WES data, showing a UPD on chromosome 9p (9p-UDP), was homozygote for the somatic *JAK2* V617F (rs77375493) mutation. Since the co-occurrence of *JAK2* V617F mutation and 9p-UPD is very common in hematological malignancies (Wang et al., 2016), a somatic (hematologic) origin for some of the reported UPD is highly conceivable, especially since 9p-UPD is the most common UPD among our UPD subjects (8/56, 14%, Figure 4).

## Discussion

Our results clearly demonstrate the effect of recent consanguinity and outbred autozygosity in increasing the risk of LOAD consistently across the eight ethnic groups analyzed, independently of *APOE*^∗^4 and EDU. Several important features separate our work from previous studies looking at the impact of consanguinity and autozygosity on LOAD. First, and most critically, this is the largest such study to date combining 11,196 cases and 10,296 controls across eight ethnically distinct populations. Second, we went beyond standard super-population definitions of ethnicity and determined European sub-ancestry (NWE, AJE, FCN, FIN, NWE, and SEE), since it has previously been established that these ethnicities have different inbreeding rates (Pemberton et al., 2012; Kang et al., 2016), or are characterized by founder effects (AJE, FCN, FIN) (Jakkula et al., 2008; Roy-Gagnon et al., 2011). Third, rather than examining autozygosity across all subjects, we enriched our sample by identifying a consanguineous subset and analyzing them separately from the relative outbred population. This step allowed us to estimate the risk for LOAD attributable to the mating types of consanguineous subjects (first cousin/double-first cousin/avuncular vs. second cousin offspring), thereby providing a measure applicable to the clinical setting. Fourth, we leveraged the large amount of WES data from ADSP to determine the contribution of rare recessive damaging variants in LOAD. Lastly, we provided, for the first time, an estimate of the impact of putative isodisomy on LOAD; these subjects were also removed from our analysis of inbred subjects, thereby eliminating a source of noise since these subjects are wrongly identified as consanguineous using standard measures.

The overall results suggest the existence of inbreeding depression, which is a recognized phenomenon that is common to polygenic traits in all living organisms (Joshi et al., 2015). Inbreeding depression is thought to result from increased homozygosity of multiple recessive alleles that act in the same direction of effect at loci that influence the phenotype of interest (“directional dominance”) (Joshi et al., 2015). In a consanguineous individual, inbreeding depression is predicted to affect many polygenic endophenotypes which can be established risk factors for late-onset diseases, such as blood pressure, body mass index, cholesterol levels, glucose levels, and bone mineral density (Rudan and Campbell, 2004). The previous negative association of educational attainment and general cognitive abilities with genome-wide autozygosity (Joshi et al., 2015) suggests involvement of directional dominance at these two endophenotypes in increasing the risk for LOAD. Indeed, it has been widely reported that lower education is associated with a greater risk for dementia (Sharp and Gatz, 2011), while lower general cognitive abilities have been linked to an increased risk of dementia according to the cognitive reserve theory (Schmand et al., 1997). However, the present results show that the association of consanguinity with LOAD is independent of educational attainment. This evidence leads us to speculate on the involvement of other polygenic endophenotypes mediating the association of consanguinity with LOAD or on the direct effect of recessive loci in LOAD, yet to be discovered. In this context, we can mention that a recent study (Andrews et al., 2021), leveraging Polygenic Risk Score/Mendelian Randomization analyses on a large sample (*N* = 26,431 LOAD cases/controls tested for 22 LOAD risk factors/clinical biomarkers), strongly supported a causal role for blood pressure and cholesterol levels with LOAD phenome. Thus, it may be conceivable that directional dominance acting on blood pressure and cholesterol levels may be contributing to the association reported here. Future studies targeting a larger subset of consanguineous subjects, phenotypically characterized in a more homogeneous way, ideally including clinically relevant biomarkers such as blood pressure and cholesterol levels, will allow us to better determine the impact of directional dominance at those endophenotypes in LOAD.

Notably, despite significant differences in consanguinity rates, autozygosity level, mean age, mean EDU, and *APOE* frequencies, each of the ethnic groups individually showed significant association (or a non-significant trend in the same direction) for the association of close consanguinity or autozygosity with an increased risk of LOAD. Our results in ACD, ECD, and YRI are reassuringly and not surprisingly, in line with the previous studies (Ghani et al., 2013, 2015) since we used overlapping datasets. However, the results in the European groups (AJE, FCN, FIN, NWE, and SEE) are new and highlight interesting differences across the five ethnicities.

Previous studies carried out on Europeans of British/Irish descent (Nalls et al., 2009a; Sims et al., 2011) reported inconsistent results on the role of ROHs in Caucasians. However, neither study had sufficient power to detect significant results given the small sample size (*N* < 3,000). Indeed, given the small variation in genome wide *F*_ROH_ in unselected samples (standard deviation in our analyses are on the order of 0.001), large sample sizes (e.g., >12,000) are necessary to detect inbreeding depression given the relatively small effect sizes in samples not selected for recent inbreeding (Keller et al., 2012).

We also leveraged WES data from ADSP to determine the contribution of rare recessive variants in LOAD. The lack of association between the global burden of rare recessive variants and LOAD suggests either the involvement of increased homozygosity at common loci or the existence of specific recessive loci driving the association of consanguinity with LOAD. The two-stage recessive-GWAS we carried out using ADSP WES data showed the association of *RPH3AL* p.A303V (rs117190076) with LOAD. The *RPH3AL* gene (also known as *NOC2*), located on 17p13.3 (OMIM^∗^604881), encodes for the Rabphilin 3A-like (without C2 domains) protein which plays an essential role in endocrine and exocrine cells, ranging from the accumulation of secretory granules of increased size to impairments in the regulated release of their secretory products (Cheviet et al., 2004). In particular, RPH3AL has been shown to be a crucial effector for RAB3A and RAB27A in the regulation of secretory vesicle exocytosis (Fukuda et al., 2004). The dysregulation of RAB3A and RAB27A has already been linked to Alzheimer’s and other neurodegenerative disorders (Davidsson et al., 2001; Ginsberg et al., 2011; Bereczki et al., 2016; Iguchi et al., 2016), while the ancestral *RPH3A* (Rabphilin 3A) gene (Craxton, 2010) was found to influence dementia severity, cholinergic deafferentation, and increased β-amyloid concentrations in postmortem neocortex of Alzheimer’s disease patients (Tan et al., 2014). Moreover, other rs117190076-unlinked variants at the *RPH3AL* locus have been associated with LOAD-related phenotypes, such as Alzheimer’s age-at-onset in *PSEN1* E280A carriers (rs4341804, *P* = 7.10 × 10^–13^) (Vélez et al., 2013) and cognitive performance scores in electronic health records (rs74192827, *P* = 5.02 × 10^–7^) (McCoy et al., 2018). Thus, the overall evidence suggests a functional role of the *RPH3AL* locus in LOAD that clearly warrants further investigations.

Interestingly, our work highlighted the presence of potential UPD carriers in the population studied, with prevalence estimates of 0.25% in NWE, 0.74% in SEE and 1.51% in ACD, respectively, showing a trend toward a significant association with increased risk of LOAD. Current estimates of UPD in the general population suggests a general prevalence of 0.05% (1 in 2,000 births) (Nakka et al., 2019), lower than the estimates we reported. One explanation for this discrepancy could be the fact that we were not able to determine whether those long, unique, ROHs (used to define the presence of UPD) may turn to be true long deleted genomic regions, of somatic or germ-line origin. Indeed, we did not have access to raw SNP-array intensity data from most of the cohorts included in our study, leading to an under-estimation of large deletions and a consequent increased number of subjects carrying a putative UPD. Nonetheless, our studied sample is mostly representative of the elderly population, where age-related somatic events, like Clonal Hematopoiesis of Indeterminate Potential (CHIP), already linked to cardiovascular disease (Jaiswal et al., 2017), may result in large somatic genomic aberrations, such as “pseudo” 9p-UPD (Wang et al., 2016), that can be misinterpreted as germline UPD. A deeper analysis of these phenomena is clearly warranted, since it may offer important insights into the missing heritability of several age-related diseases. In this context, it is remarkable that functional mutations of the *TET2* gene, a main driver of CHIP (Jaiswal et al., 2017), have recently been found to be associated with multiple neurodegenerative disorders, including LOAD (Cochran et al., 2020).

One important limitation is that the ethnic stratification, especially for the European groups, led to very small samples in terms of the number of inbred subjects for some of the ancestral groups [e.g., FCN, *N* = 53; FIN, *N* = 31 (Supplementary Tables S6–S13)]. Nonetheless, the meta-analytic approach used can greatly mitigate potential biases due to the inclusions of small samples, while providing a better sense of the ethnic-related differences in consanguinity prevalence. Similarly, considering the important contribution of somatic genomic events related to aging such as CHIP, the heterogeneous nature of the specimens used (e.g., whole blood vs. post-mortem brain tissue) in SNP-array genotyping across the different cohorts and samples may have led to uncontrolled biases when determining the impact of true ROHs vs. large genomic deletions of somatic origin.

Overall, these results provide substantial evidence that consanguinity increases risk for LOAD. One might anticipate a change in the genetic architecture of LOAD in the coming decades when more recent cohorts, composed of subjects born after the World War II, will be analyzed. Panmixia and larger effective population sizes have resulted in decreasing autozygosity as the chronological age of a population decreases (Nalls et al., 2009b). Consistent with this pattern, mounting evidence suggests that trends in dementia incidence rates are decreasing (Satizabal et al., 2016; Derby et al., 2017; Noble et al., 2017). Subsequent work with increased sample sizes of consanguineous subjects should accelerate the discovery of non-additive genetic effects in LOAD.

## Alzheimer’s Disease Neuroimaging Initiative

Data used in preparation of this article were obtained from the Alzheimer’s Disease Neuroimaging Initiative (ADNI) database (adni.loni.usc.edu). As such. the investigators within the ADNI contributed to the design and implementation of ADNI and/or provided data but did not participate in analysis or writing of this report. A complete listing of ADNI investigators can be found at: http://adni.loni.usc.edu/wp-content/uploads/how_to_apply/ADNI_Acknowledgement_List.pdf.

## Data Availability Statement

The original contributions presented in the study are included in the article/Supplementary Material, further inquiries can be directed to the corresponding author/s.

## Ethics Statement

This was a re-analysis of de-identified data available from shared data repositories. The study protocol was granted an exemption by the Stanford Institutional Review Board because the analyses were carried out on “de-identified, off-the-shelf” data. The patients/participants provided their written informed consent to participate in this study.

## Author Contributions

VN: conceptualization, data curation, formal analysis, investigation, methodology, and writing – original draft preparation. MS: formal analysis, validation, and writing – review and editing. RK: formal analysis, resources, writing – review and editing. AA: writing – review and editing. MG: funding acquisition, supervision, and writing – review and editing. All authors contributed to the article and approved the submitted version.

## Conflict of Interest

The authors declare that the research was conducted in the absence of any commercial or financial relationships that could be construed as a potential conflict of interest.
