# Supplementary material for: Recent Consanguinity and Outbred Autozygosity Are Associated With Increased Risk of Late-Onset Alzheimer’s Disease
Source: Front Genet. 2021 Jan 29;11:629373. doi: 10.3389/fgene.2020.629373 (PMC7879576; doi:10.3389/fgene.2020.629373)

**Table S1. Participating studies**. Genotyping platform used. human genome build. number of subjects and dataset repository.

| **DATASET** | **Genotyping Platform** | **hg** | **N** | **Dataset Repository** |
| --- | --- | --- | --- | --- |
| ACT | Illumina Human 660W-Quad | 19 | 2789 | NIAGADS/dbGAP |
| ADC1 | Illumina Human 660W-Quad | 18 | 2115 | NIAGADS |
| ADC2 | Illumina Human 660W-Quad | 18 | 928 | NIAGADS |
| ADC3 | Illumina Human OmniExpress | 18 | 1526 | NIAGADS |
| ADDNEUROMED | Illumina Human 610-Quad  Illumina Human OmniExpress | 19  19 | 329  315 | Synapse |
|  | Illumina Omni 2.5 | 19 | 800 | ADNI |
| ADNI | Illumina Human 610-Quad | 18 | 490 |  |
|  | Illumina Human OmniExpress | 19 | 357 |  |
| Columbia University Study of Caribbean Hispanics with  Familial and Sporadic Late Onset Alzheimer's disease (CIDR) | Illumina Human Omni1-Quad | 19 | 3064 | dbGAP |
| GenADA | Affymetrix 500K | 18 | 1577 | dbGAP |
| HBTRC | Illumina Human Hap650Y | 19 | 740 | Synapse |
| Indianapolis-Ibadan Dementia Project (IIDP) | Illumina Omni 2.5 | 19 | 1251 | dbGAP |
| NIA-LOAD | Illumina Human 610-Quad | 18 | 5220 | NIAGADS |
| MAYO | Illumina Human Hap300 | 18 | 2099 | NIAGADS |
| MIRAGE | Illumina Human CNV370-Duo | 18 | 397 | NIAGADS |
|  | Illumina Human 610-Quad | 18 | 1105 |  |
| OHSU | Illumina Human CNV370-Duo | 18 | 647 | NIAGADS |
| ROSMAP | Affymetrix 6.0 | 18 | 1703 | Synapse |
| ROSMAP2 | Illumina Human OmniExpress | 19 | 382 | Synapse |
| TGEN | Affymetrix 6.0 | 19 | 1599 | NIAGADS |
| UPITT | Illumina Human Omni1-Quad | 18 | 2440 | NIAGADS |
| UMVUMSSM | Illumina Human 550K. Illumina Human 610-Quad | 19 | 680 | NIAGADS |
|  | Illumina Human 1M-Duo | 19 | 681 |  |
|  | Affymetrix 6.0 | 19 | 448 |  |
| WASHU | Illumina Human 610-Quad | 18 | 670 | NIAGADS |
|  | **TOTAL** |  | 34352 |  |

**Table S2. QC processed sample demographics in the 20 GWAS datasets.** Subjects showing sex inconsistency, autosome missingness (>5%), contamination and missing/other phenotype were excluded from individual ancestry determination. EUR=European; AFR=African; EAS=East-Asian; AMR=American; SAS=South-East Asian. NA=Not Available

|  | **ACT** | **ADC1** | **ADC2** | **ADC3** | **ADDNEUROMED** | **ADNI** | **CIDR** | **GenADA** | **HBTRC** | **IIDP** | **NIA-LOAD** | | **MAYO** | **MIRAGE** | **OHSU** | **ROSMAP** | **ROSMAP2** | **TGEN** | **UPITT** | **UMVUMSSM** | **WASHU** | **TOTAL** |
| --- | --- | --- | --- | --- | --- | --- | --- | --- | --- | --- | --- | --- | --- | --- | --- | --- | --- | --- | --- | --- | --- | --- |
| **TOTAL** | 2789 | 2115 | 928 | 1526 | 644 | 1647 | 3064 | 1577 | 740 | 1251 | 5220 | | 2099 | 1502 | 647 | 1703 | 382 | 1599 | 2440 | 1809 | 670 | 34352 |
| **PHENOTYPE QC** | | | | | | | | | | | | | | | | | | | | | | |
| **MISSING**  **STATUS/**  **non-AD** | 446 | 144 | 181 | 270 | 204 | 556 | 12 | 0 | 306 | 43 | 670 | | 1 | 15 | 221 | 403 | 103 | 0 | 88 | 0 | 104 | 3767 |
| **MISSING**  **AGE/**  **AGE<60yrs** | 1 | 1 | 4 | 124 | 8 | 29 | 137 | 95 | 87 | 0 | 544 | | 0 | 237 | 2 | 1 | 0 | 54 | 11 | 147 | 26 | 1508 |
| **1^st^ GENOTYPE QC** | | | | | | | | | | | | | | | | | | | | | | |
| **Autosomes Missingness** | 0 | 0 | 0 | 0 | 0 | 0 | 0 | 6 | 1 | 0 | 0 | | 55 | 0 | 3 | 105 | 0 | 75 | 72 | 0 | 0 | 317 |
| **X-Chr.**  **Missingness** | 2 | 6 | 0 | 0 | 2* | 13 | 0 | 9 | NA** | 3 | 33 | | 24 | 22 | 2 | 13 | 0 | 140 | 88 | 4 | 3 | 364 |
| **1^st^ ETHNIC DETERMINATION** | | | | | | | | | | | | | | | | | | | | | | |
| **EUR** | 2140 | 1961 | 740 | 1121 | 421 | 958 | 99 | 1464 | 345 | 0 | 3370 | 1987 | | 1199 | 416 | 1180 | 279 | 1320 | 2168 | 1640 | 537 | 23345 |
| **AFR** | 30 | 0 | 0 | 0 | 0 | 17 | 50 | 0 | 0 | 1205 | 61 | 3 | | 1 | 0 | 0 | 0 | 0 | 4 | 4 | 0 | 1375 |
| **EAS** | 68 | 0 | 0 | 0 | 0 | 17 | 0 | 0 | 0 | 0 | 8 | 5 | | 0 | 0 | 0 | 0 | 0 | 0 | 0 | 0 | 98 |
| **AMR** | 6 | 0 | 0 | 0 | 0 | 10 | 65 | 2 | 0 | 0 | 2 | 0 | | 2 | 0 | 0 | 0 | 1 | 1 | 0 | 0 | 89 |
| **SAS** | 0 | 0 | 0 | 0 | 0 | 3 | 0 | 0 | 0 | 0 | 0 | 0 | | 0 | 0 | 0 | 0 | 1 | 0 | 0 | 0 | 4 |
| **ADMIXED** | 96 | 3 | 3 | 11 | 9 | 44 | 2701 | 1 | 1 | 0 | 532 | 24 | | 26 | 3 | 1 | 0 | 8 | 8 | 14 | 0 | 3489 |

*X-chromosome data not available for ADDNEUROMED (OmniExpress) subjects

** X-chromosome data not available

**Table S3. Estimated individual ancestry in the European samples.** Studied samples of European ancestry were further stratified according to their percentage of Northwestern (NWE), Southeastern (SEE), Ashkenazi Jewish (AJE), French-Canadian (FCN) and Finnish (FIN) European ancestry.

|  | **ACT** | **ADC1** | **ADC2** | **ADC3** | **ADDNEUROMED** | **ADNI** | **GenADA** | **HBTRC** | **MAYO** | **MIRAGE** | **NIA-LOAD** | **OHSU** | **ROSMAP** | **ROSMAP2** | **TGEN** | **UPITT** | **UMVUMSSM** | **WASHU** | **TOTAL** |
| --- | --- | --- | --- | --- | --- | --- | --- | --- | --- | --- | --- | --- | --- | --- | --- | --- | --- | --- | --- |
| **TOTAL** | 2140 | 1961 | 740 | 1121 | 421 | 958 | 1464 | 345 | 1987 | 1199 | 3370 | 416 | 1180 | 279 | 1320 | 2168 | 1640 | 537 | 23246 |
|  | | | | | | | | | | | | | | | | | | | |
| **NWE** | 1919 | 1703 | 617 | 944 | 131 | 810 | 995 | 302 | 1767 | 820 | 3025 | 395 | 1111 | 270 | 1188 | 1673 | 1266 | 477 | 19413 |
| **SEE** | 51 | 65 | 38 | 43 | 181 | 35 | 10 | 17 | 41 | 265 | 112 | 5 | 26 | 3 | 33 | 308 | 66 | 12 | 1311 |
| **AJE** | 104 | 164 | 67 | 94 | --- | 74 | 58 | 6 | 44 | 47 | 185 | 10 | 10 | 1 | 69 | 128 | 270 | 35 | 1366 |
| **FCN** | --- | --- | --- | --- | --- | --- | 381 | --- | --- | --- | --- | --- | --- | --- | --- | --- | --- | --- | 381 |
| **FIN** | 37 | 6 | 5 | 8 | 105 | 6 | 5 | 5 | 19 | 1 | 6 | 3 | 6 | 1 | 3 | 2 | 7 | 1 | 226 |
|  | | | | | | | | | | | | | | | | | | | |
| **ADMIXED** | 29 | 23 | 13 | 32 | 4 | 33 | 15 | 15 | 116 | 66 | 42 | 3 | 27 | 4 | 27 | 57 | 31 | 12 | 549 |

**Table S4. GWAS grouping according to SNP-array manufacturer’.** GWAS grouping was performed to maximize the number of shared SNPs for QC procedures and imputation.

| **Group** | **1** | **2** | **3** | **4** | **5** | **6** |
| --- | --- | --- | --- | --- | --- | --- |
|  | ACT | ADC3 | ROSMAP | MAYO | GenADA* | HBTRC |
|  | ADC1 | ADDNEUROMED (OmniExpress) | TGEN | MIRAGE (CNV370-Duo) |  |  |
|  | ADC2 | ADNI (OmniExpress) | UVM (Affymetrix 6.0) | OHSU |  |  |
|  | ADDNEUROMED (610-Quad) | ADNI (Omni 2.5) |  |  |  |  |
|  | ADNI (610-Quad) | ROSMAP2 |  |  |  |  |
|  | MIRAGE (610-Quad) | UPITT |  |  |  |  |
|  | NIA-LOAD | **CIDR |  |  |  |  |
|  | UVM_A | ***IIDP |  |  |  |  |
|  | UVM_B |  |  |  |  |  |
|  | WASHU |  |  |  |  |  |
| **ARRAY** | ILLUMINA | ILLUMINA | AFFYMETRIX | ILLUMINA | AFFYMETRIX | ILLUMINA |
|  |  | | | | | |
| **NWE (N)** | 7762 | 3181 | 2301 | 2020 | 995 | 302 |
| **AJE (N)** | 754 | 243 | 113 | 57 | 57 | 6 |
| **SEE (N)** | 515 | 385 | 59 | 65 | 10 | 17 |
| **FCN (N)** | --- | --- | --- | --- | 381 | --- |
| **FIN (N)****** | 138 | 59 | 16 | 21 | 7 | 5 |
| **YRI (N)** | --- | 1205 | --- | --- | --- | --- |
| **ECD (N)** | --- | 999 | --- | --- | --- | --- |
| **ACD (N)** | --- | 446 | --- | --- | --- | --- |

*GenADA NWE, AJE, SEE and FIN subjects were QCed and imputed in a single batch, without considering their ethnicity.

**CIDR subjects where divided into two ethnic groups European-Caribbean (ECD) and African-Caribbean (ACD), respectively (as reported in Materials and Methods), and processed independently.

**IIDP includes only African subjects. It was not included in the determination of final shared SNPs, and it was QCed along with the other African samples.

****FIN samples were QCed and imputed along with NWE given their small sample size.

**Table S5. Autosomal SNPs used for imputation and number of final SNPs used for GWAS after QC in the twenty GWAS datasets.** *French-Canadians (FCN) and Finnish Europeans (FIN) were imputed and QCed along with NWE subjects.

|  | **NWE** | | **AJE** | | **SEE** | | **FCN*** | | **FIN*** | | **AFR** | | **EUR-CAR** | | **AFR-CAR** | |
| --- | --- | --- | --- | --- | --- | --- | --- | --- | --- | --- | --- | --- | --- | --- | --- | --- |
| **GWAS GROUP** | **Final Shared SNPs** | **Imputed SNPs (r2>0.7)** | **Final Shared SNPs** | **Imputed SNPs (r2>0.7)** | **Final Shared SNPs** | **Imputed SNPs (r2>0.7)** | **Final Shared SNPs** | **Imputed SNPs (r2>0.7)** | **Final Shared SNPs** | **Imputed SNPs (r2>0.7)** | **Final Shared SNPs** | **Imputed SNPs (r2>0.7)** | **Final Shared SNPs** | **Imputed SNPs**  **(r2>0.7)** | **Final Shared SNPs** | **Imputed SNPs**  **(r2>0.7)** |
| 1 | 450,495 | 15,984,530 | 446,056 | 12,068,990 | 443,736 | 10,010,446 | --- | --- | 450,495 | 15,984,530 | --- | --- | --- | --- | --- | --- |
| 2 | 525,644 | 14,233,131 | 522,412 | 10,993,082 | 524,783 | 10,607,381 | --- | --- | 525,644 | 14,233,131 | 586,214 | 15,420,541 | 855,518 | 10,567,858 | 855,763 | 12,719,705 |
| 3 | 394,712 | 11,472,180 | 390,538 | 9,319,737 | 391,408 | 6,861,750 | --- | --- | 394,712 | 11,472,180 | --- | --- | --- | --- | --- | --- |
| 4 | 289,789 | 12,017,210 | 289,782 | 8,383,541 | 289,314 | 7,389,782 | --- | --- | 289,789 | 12,017,210 | --- | --- | --- | --- | --- | --- |
| 5 | 307,048 | 9,170,603 | 307,048 | 9,170,603 | 307,048 | 9,170,603 | 307,048 | 9,170,603 | 307,048 | 9,170,603 | --- | --- | --- | --- | --- | --- |
| 6 | 533,598 | 39,127,692 | 533,598 | 39,127,692 | 533,598 | 39,127,692 | --- | --- | 533,598 | 39,127,692 | --- | --- | --- | --- | --- | --- |

**Table S6. Merged North-Western European (NWE) samples.**

|  | **OUTBRED** | **INBRED** | **Mean Age**  **OUTBRED** | **Mean Age**  **INBRED** | **Age Difference P-value** | ****Mean EDU OUTBRED** | ****Mean EDU**  **INBRED** | **EDU Difference P-value** |
| --- | --- | --- | --- | --- | --- | --- | --- | --- |
| **TOTAL** | 16820 | 613 |  |  |  |  |  |  |
| Duplicates | 545 | 10 |  |  |  |  |  |  |
| Related* | 277 | 63 |  |  |  |  |  |  |
| Putative isodisomy | 42 | |  |  |  |  |  |  |
| **TOTAL** | 15996 (97.0%) | 500 (3.0%) | 76.2±8.3 | 76.0±8.6 | 0.526 | 15.2±3.3 | 14.0±4.1 | **6.1x10^-6^** |
| Cases | 8557 (96.5%) | 313 (3.5%) | 74.7±8.1 | 74.9±8.4 | 0.628 | 14.8±3.4 | 13.3±4.3 | **1.2x10^-4^** |
| Controls | 7439 (97.5%) | 187 (2.5%) | 78.0±8.2 | 77.9±8.6 | 0.765 | 15.5±3.2 | 14.7±3.7 | **0.045** |
| Males | 6490 (97.1%) | 193 (2.9%) | 75.7±8.0 | 75.2±8.6 | 0.532 | 15.9±3.5 | 15.5±3.6 | 0.413 |
| Females | 9506 (96.9%) | 307 (3.1%) | 76.6±8.5 | 76.5±8.6 | 0.701 | 14.8±3.1 | 13.1±4.1 | **<1x10^-7^** |

*Inter-dataset related (PI-Hat>0.0625)

** ”EDU (Years of Education)” variable available for 6188 subjects (37.5% of the total sample)

**Table S7. Merged Ashkenazi-Jewish European (AJE) samples.**

|  | **OUTBRED** | **INBRED** | **Mean Age OUTBRED** | **Mean Age**  **INBRED** | **Age Difference P-value** | ****Mean EDU OUTBRED** | ****Mean EDU**  **INBRED** | **EDU Difference P-value** |
| --- | --- | --- | --- | --- | --- | --- | --- | --- |
| **TOTAL** | 1366 | |  |  |  |  |  |  |
| Sex Inconsistency | 4 | --- |  |  |  |  |  |  |
| Discordant Ancestry | 7 | --- |  |  |  |  |  |  |
| Duplicates | 22 | 4 |  |  |  |  |  |  |
| Related* | 95 | 5 |  |  |  |  |  |  |
| Putative isodisomy | --- | |  |  |  |  |  |  |
| **TOTAL** | 1088 (88.5%) | 141 (11.5%) | 78.4±7.9 | 79.2±8.1 | 0.292 | 16.3±2.9 | 16.4±2.9 | 0.677 |
| Cases | 674 (89.4%) | 80 (10.6%) | 77.9±7.6 | 78.2±7.6 | 0.814 | 15.4±3.1 | 16.1±2.7 | 0.309 |
| Controls | 414 (87.2%) | 61 (12.8%) | 79.2±8.4 | 80.5±8.7 | 0.259 | 16.9±2.7 | 16.7±3.0 | 0.712 |
| Males | 464 (88.1%) | 63 (11.9%) | 77.7±7.6 | 78.2±7.5 | 0.671 | 17.1±3.0 | 17.6±2.7 | 0.433 |
| Females | 624 (88.9%) | 78 (11.1%) | 78.9±8.2 | 80.0±8.6 | 0.287 | 15.5±2.6 | 15.5±2.7 | 0.929 |

*Inter-dataset related (PI-Hat>0.0625)

** ”Years of Education” variable available for 433 subjects (35.2% of the total sample)

**Table S8. Merged South-Eastern European (SEE) samples.**

|  | **OUTBRED** | **INBRED** | **Mean Age OUTBRED** | **Mean Age**  **INBRED** | | **Age Difference P-value** | | ****Mean EDU OUTBRED** | ****Mean EDU**  **INBRED** | | **EDU Difference P-value** | |
| --- | --- | --- | --- | --- | --- | --- | --- | --- | --- | --- | --- | --- |
| **TOTAL** | 1311 | |  | |  | |  |  | |  | |  |
| Sex Inconsistency | 3 | --- |  |  | |  | |  |  | |  | |
| Discordant Ancestry | 35 | --- |  |  | |  | |  |  | |  | |
| Duplicates | 21 | 4 |  |  | |  | |  |  | |  | |
| Related* | 128 | 29 |  |  | |  | |  |  | |  | |
| Putative isodisomy | 8 | |  |  | |  | |  |  | |  | |
| **TOTAL** | 899 (83.0%) | 184 (17.0%) | 74.5±7.5 | 75.0±7.2 | | 0.424 | | 11.1±6.3 | 11.4±4.6 | | 0.749 | |
| Cases | 562 (82.9%) | 116 (17.1%) | 73.8±7.2 | 73.8±6.9 | | 0.969 | | 9.9±5.2 | 10.8±4.9 | | 0.311 | |
| Controls | 337 (83.2%) | 68 (16.8%) | 75.7±7.8 | 77.0±7.3 | | 0.183 | | 12.6±7.2 | 12.4±3.8 | | 0.918 | |
| Males | 371 (82.6%) | 78 (17.4%) | 74.2±7.3 | 74.2±6.6 | | 0.935 | | 12.6±7.5 | 12.3±5.3 | | 0.828 | |
| Females | 528 (83.3%) | 106 (16.7%) | 74.7±7.6 | 75.6±7.5 | | 0.268 | | 10.0±5.1 | 10.6±3.9 | | 0.567 | |

*Inter-dataset related (PI-Hat>0.0625)

**” Years of Education” variable available for 400 subjects (36.9% of the total sample)

**Table S9. Merged French-Canadian European samples.**

|  | **OUTBRED** | **INBRED** | **Mean Age OUTBRED** | **Mean Age**  **INBRED** | |
| --- | --- | --- | --- | --- | --- |
| **TOTAL** | 381 | |  | |  |
| Sex Inconsistency | --- | --- |  |  | |
| Discordant Ancestry | --- | --- |  |  | |
| Duplicates | --- | --- |  |  | |
| Related* | 2 | 1 |  |  | |
| Putative isodisomy | --- | |  |  | |
| **TOTAL** | 323 (85.9%) | 53 (14.1%) | 74.1±7.0 | 75.3±7.1 | |
| Cases | 164 (85.0%) | 29 (15.0%) | 73.8±7.1 | 76.1±7.3 | |
| Controls | 159 (86.9%) | 24 (13.1%) | 74.3±6.9 | 74.3±6.8 | |
| Males | 85 (85.0%) | 15 (15.0%) | 74.7±6.4 | 75.3±6.5 | |
| Females | 238 (86.2%) | 38 (13.8%) | 73.9±7.2 | 75.2±7.4 | |

*Inter-dataset related (PI-Hat>0.0625)

**Table S10. Merged Finnish European (FIN) samples.**

|  | **OUTBRED** | **INBRED** | **Mean Age OUTBRED** | **Mean Age**  **INBRED** | **Age Difference**  **P-value** | ****Mean EDU OUTBRED** | ****Mean EDU**  **INBRED** | **EDU Difference**  **P-value** |
| --- | --- | --- | --- | --- | --- | --- | --- | --- |
| **TOTAL** | 226 | |  |  |  |  |  |  |
| Sex Inconsistency | --- | --- |  |  |  |  |  |  |
| Discordant Ancestry | --- | --- |  |  |  |  |  |  |
| Duplicates | 4 | --- |  |  |  |  |  |  |
| Related* | 3 | --- |  |  |  |  |  |  |
| Putative isodisomy | --- | |  |  |  |  |  |  |
| **TOTAL** | 188 (85.8%) | 31 (14.2%) | 76.3±7.6 | 76.8±6.8 | 0.770 | 10.8±4.5 | 9.2±3.7 | 0.080 |
| Cases | 92 (83.6%) | 18 (16.4%) | 74.3±8.2 | 74.4±6.0 | 0.984 | 10.1±4.4 | 8.0±3.3 | 0.082 |
| Controls | 96 (88.1%) | 13 (11.9%) | 78.3±6.4 | 80.1±6.6 | 0.343 | 11.4±4.5 | 11.0±3.6 | 0.797 |
| Males | 78 (84.8%) | 14 (15.2%) | 75.2±7.1 | 76.7±7.2 | 0.478 | 11.1±4.9 | 9.6±3.9 | 0.317 |
| Females | 110 (86.6%) | 17 (13.4%) | 77.1±7.8 | 76.9±6.7 | 0.876 | 10.6±4.2 | 8.7±3.4 | 0.124 |

*Inter-dataset related (PI-Hat>0.0625)

** ”Years of Education” variable available for 159 subjects (72.6% of the total sample)

**Table S11. European-Caribbean from Dominican Republic (ECD) samples.**

|  | **OUTBRED** | **INBRED** | **Mean Age OUTBRED** | **Mean Age**  **INBRED** | **Age Difference P-value** | **Mean EDU OUTBRED** | **Mean EDU**  **INBRED** | **EDU Difference P-value** |
| --- | --- | --- | --- | --- | --- | --- | --- | --- |
| **TOTAL** | 1037 | |  |  |  |  |  |  |
| Sex Inconsistency | --- | --- |  |  |  |  |  |  |
| Discordant Ancestry | --- | --- |  |  |  |  |  |  |
| Duplicates | --- | --- |  |  |  |  |  |  |
| Related* | 366 | |  |  |  |  |  |  |
| Putative isodisomy | --- | |  |  |  |  |  |  |
| **TOTAL** | 458 (68.3%) | 213 (31.7%) | 74.6±8.0 | 74.0±8.1 | 0.318 | 7.0±5.1 | 5.6±4.6 | **0.0005** |
| Cases | 220 (65.3%) | 117 (34.7%) | 74.9±7.6 | 74.7±7.7 | 0.851 | 5.3±4.5 | 4.0±3.7 | **0.006** |
| Controls | 238 (71.3%) | 96 (28.7%) | 74.4±8.4 | 73.0±8.5 | 0.184 | 8.6±5.2 | 7.5±5.0 | 0.077 |
| Males | 139 (63.2%) | 81 (36.8%) | 75.1±8.0 | 73.9±9.1 | 0.331 | 7.6±5.7 | 5.7±4.8 | **0.012** |
| Females | 319 (70.7%) | 132 (29.3%) | 74.4±8.0 | 74.0±7.5 | 0.579 | 6.8±4.9 | 5.5±4.6 | **0.010** |

*Inter-dataset related (PI-Hat>0.0625)

**Table S12. African-Caribbean from Dominican Republic (ACD) samples.**

|  | **OUTBRED** | **INBRED** | **Mean Age OUTBRED** | **Mean Age**  **INBRED** | **Age Difference P-value** | **Mean EDU OUTBRED** | **Mean EDU**  **INBRED** | **EDU Difference P-value** |
| --- | --- | --- | --- | --- | --- | --- | --- | --- |
| **TOTAL** | 457 | |  |  |  |  |  |  |
| Sex Inconsistency | 1 | --- |  |  |  |  |  |  |
| Discordant Ancestry | --- | --- |  |  |  |  |  |  |
| Duplicates | --- | --- |  |  |  |  |  |  |
| Related* | 52 | |  |  |  |  |  |  |
| Putative isodisomy | 6 | |  |  |  |  |  |  |
| **TOTAL** | 304 (76.4%) | 94 (23.6%) | 74.7±8.1 | 73.8±7.0 | 0.181 | 5.3±4.9 | 4.0±4.0 | **0.015** |
| Cases | 123 (72.3%) | 47 (27.7%) | 76.7±7.4 | 73.9±7.4 | **0.020** | 3.0±3.4 | 2.3±2.4 | 0.271 |
| Controls | 181 (79.4%) | 47 (20.6%) | 73.3±8.3 | 73.7±6.7 | 0.915 | 6.9±5.2 | 5.8±4.5 | 0.103 |
| Males | 88 (72.7%) | 33 (27.3%) | 74.6±7.9 | 72.3±5.6 | **0.039** | 5.3±4.8 | 4.4±3.0 | 0.245 |
| Females | 216 (78.0%) | 61 (22.0%) | 74.7±8.2 | 74.6±7.6 | 0.862 | 5.3±4.9 | 3.8±4.4 | **0.031** |

*Inter-dataset related (PI-Hat>0.0625)

**Table S13. African-Yoruba (YRI) samples.**

|  | **OUTBRED** | **INBRED** | **Mean Age**  **OUTBRED** | | **Mean Age**  **INBRED** | **Age Difference P-value** | **Mean EDU OUTBRED** | **Mean EDU**  **INBRED** | **EDU Difference P-value** |
| --- | --- | --- | --- | --- | --- | --- | --- | --- | --- |
| **TOTAL** | 1205 | |  |  | |  |  | |  |
| Sex Inconsistency | 1 | --- |  | |  |  |  |  |  |
| Discordant Ancestry | --- | --- |  | |  |  |  |  |  |
| Duplicates | --- | --- |  | |  |  |  |  |  |
| Related* | 184 | --- |  | |  |  |  |  |  |
| Putative isodisomy | --- | |  | |  |  |  |  |  |
| **TOTAL SUBJECTS** | 1008 (98.8%) | 12 (1.2%) | 83.1±6.2 | | 81.2±5.5 | 0.279 | 0.7±2.2 | 2.8±3.9 | **0.002** |
| **Cases** | 83 (98.8%) | 1 (1.2%) | 84.5±8.0 | | 85 | 0.951 | 0.4±2.0 | 6.0 | **0.007** |
| **Controls** | 925 (98.8%) | 11 (1.2%) | 83.0±6.0 | | 80.8±5.6 | 0.233 | 0.7±2.3 | 2.5±4.0 | **0.012** |
| **Males** | 342 (98.6%) | 5 (1.4%) | 83.5±6.6 | | 83.4±7.1 | 0.973 | 1.5±3.2 | 5.0±4.9 | **0.017** |
| **Females** | 666 (99.0%) | 7 (1.0%) | 82.9±6.0 | | 79.6±3.8 | 0.140 | 0.3±1.4 | 1.1±2.3 | **0.091** |

*Inter-dataset related (PI-Hat>0.0625)

**Table S14. *APOE* distribution in [A] North-Western European (NWE) [B] Ashkenazi Jewish (AJE) [C] South-Eastern (SEE) European [D] French-Canadians (FCN) [E] Finns (FIN) [F] Yoruba-Africans (YRI) [G] European-Caribbean from Dominican Republic and [H] African-Caribbean from Dominican Republic samples.** Significant results are reported in bold.

A)

| **NWE** | ***APOE* genotype** | | | | | | ***APOE* allele** | | |  |  |  |
| --- | --- | --- | --- | --- | --- | --- | --- | --- | --- | --- | --- | --- |
| **STATUS** | 2/2 | 2/3 | 2/4 | 3/3 | 3/4 | 4/4 | 2* | 3* | 4* | HWE-P | Genotype | Allele |
| AD outbred (N=8551) | 12 (0.1%) | 328 (3.8%) | 256 (3.0%) | 2857 (33.4%) | 3887  (45.5%) | 1211 (14.2%) | 608 (3.6%) | 9929 (58.0%) | 6565  (38.4%) | 0.556 | 0.549 | 0.462 |
| AD inbred (N=314) | --- | 12 (3.8%) | 8 (2.6%) | 91 (29.0%) | 158 (50.3%) | 45 (14.3%) | 20 (3.2%) | 352 (56.0%) | 256  (40.8%) | 0.835 |  |  |
| AD Total (N=8865) | 12 (0.1%) | 340 (3.8%) | 264 (3.0%) | 2948 (33.3%) | 4045 (45.6%) | 1256 (14.2%) | 628 (3.5%) | 10281 (58.0%) | 6821 (38.5%) | 0.509 |  |  |
| CTL outbred (N=7431) | 58 (0.8%) | 992 (13.4%) | 148 (2.0%) | 4659  (62.7%) | 1483 (20.0%) | 91 (1.2%) | 1256 (8.5%) | 11793  (79.3%) | 1813  (12.2%) | 0.700 | 0.183 | 0.097 |
| CTL inbred (N=186) | 3  (1.6%) | 23 (12.4%) | 6 (3.2%) | 107 (57.5%) | 42 (22.6%) | 5  (2.7%) | 35  (9.4%) | 279 (75.0%) | 58 (15.6%) | 0.991 |  |  |
| CTL Total (N=7617) | 61 (0.8%) | 1015 (13.3%) | 154 (2.0%) | 4766 (62.6%) | 1525 (20.0%) | 96 (1.3%) | 1291 (8.5%) | 12072 (79.2%) | 1871 (12.3%) | 0.741 |  |  |
| TOTAL (N=16482) | 73 (0.4%) | 1355  (8.2%) | 418 (2.5%) | 7714 (46.8%) | 5570  (33.8%) | 1352 (8.2%) | 1919  (5.8%) | 22353  (67.8%) | 8692  (26.4%) |  |  |  |

B)

| **AJE** | ***APOE* genotype** | | | | | | ***APOE* allele** | | |  |  |  |
| --- | --- | --- | --- | --- | --- | --- | --- | --- | --- | --- | --- | --- |
| **STATUS** | 2/2 | 2/3 | 2/4 | 3/3 | 3/4 | 4/4 | 2* | 3* | 4* | HWE-P | Genotype | Allele |
| AD outbred (N=674) | 3  (0.5%) | 33 (4.9%) | 18 (2.7%) | 266 (39.5%) | 295 (43.8%) | 59 (8.8%) | 57 (4.2%) | 860 (63.8%) | 431  (32.0%) | 0.741 | 0.081 | 0.171 |
| AD inbred (N=80) | 1  (1.3%) | 3  (3.8%) | 1 (1.3%) | 44 (55.0%) | 23 (28.8%) | 8  (10.0%) | 6  (3.8%) | 114 (71.2%) | 40 (25.0%) | 0.736 |  |  |
| AD Total (N=754) | 4 (0.5%) | 36 (4.8%) | 19 (2.5%) | 310 (41.1%) | 318 (42.2%) | 67 (8.9%) | 63 (4.2%) | 974 (64.6%) | 471 (31.2%) | 0.737 |  |  |
| CTL outbred (N=414) | 6  (1.5%) | 53 (12.8%) | 11 (2.7%) | 258  (62.3%) | 83 (20.1%) | 3  (0.7%) | 76 (9.2%) | 652 (78.7%) | 100 (12.1%) | 0.829 | **0.030** | **0.005** |
| CTL inbred (N=61) | --- | 10 (16.4%) | --- | 48 (78.7%) | 3  (4.9%) | --- | 10  (8.2%) | 109 (89.3%) | 3  (2.5%) | 0.969 |  |  |
| CTL Total (N=475) | 6 (1.3%) | 63 (13.3%) | 11 (2.3%) | 306 (64.4%) | 86 (18.1%) | 3 (0.6%) | 86 (9.1%) | 761 (80.1%) | 103 (10.8%) | 0.853 |  |  |
| TOTAL (N=1229) | 10 (0.8%) | 99 (8.1%) | 30 (2.4%) | 616 (50.1%) | 404 (32.9%) | 70 (5.7%) | 149 (6.1%) | 1735 (70.6%) | 574 (23.3%) |  |  |  |

C)

| **SEE** | ***APOE* genotype** | | | | | | ***APOE* allele** | | |  |  |  |
| --- | --- | --- | --- | --- | --- | --- | --- | --- | --- | --- | --- | --- |
| **STATUS** | 2/2 | 2/3 | 2/4 | 3/3 | 3/4 | 4/4 | 2* | 3* | 4* | HWE-P | Genotype | Allele |
| AD outbred (N=561) | --- | 17 (3.1%) | 15 (2.7%) | 286  (51.0%) | 208 (37.1%) | 35 (6.2%) | 32  (2.9%) | 797  (71.0%) | 293 (26.1%) | 0.530 | 0.318 | 0.820 |
| AD inbred (N=115) | --- | 7  (6.1%) | 1 (0.9%) | 56 (48.7%) | 46 (40.0%) | 5  (4.4%) | 8  (3.5%) | 165  (71.7%) | 57  (24.8%) | 0.893 |  |  |
| AD Total (N=676) | --- | 24 (3.6%) | 16 (2.4%) | 342 (50.6%) | 254 (37.6%) | 40 (5.9%) | 40 (3.0%) | 962 (71.1%) | 350 (25.9%) | 0.783 |  |  |
| CTL outbred (N=337) | --- | 34 (10.1%) | 3  (0.9%) | 249 (73.9%) | 44 (13.1%) | 7  (2.1%) | 37 (5.5%) | 576 (85.5%) | 61  (9.0%) | 0.647 | 0.979 | 0.964 |
| CTL inbred (N=68) | --- | 7 (10.3%) | 1 (1.5%) | 49 (72.1%) | 10 (14.7%) | 1  (1.5%) | 8  (5.9%) | 115 (84.6%) | 13  (9.5%) | 1.000 |  |  |
| CTL Total (N=405) | --- | 41 (10.1%) | 4 (1.0%) | 298 (73.6%) | 54 (13.3%) | 8 (2.0%) | 45 (5.6%) | 691 (85.3%) | 74 (9.1%) | 0.545 |  |  |
| TOTAL (N=1081) | --- | 65 (6.0%) | 20 (1.9%) | 640 (59.2%) | 308  (28.5%) | 48  (4.4%) | 85 (3.9%) | 1653 (76.5%) | 424  (19.6%) |  |  |  |

D)

| **FCN** | ***APOE* genotype** | | | | | | ***APOE* allele** | | |  |  |  |
| --- | --- | --- | --- | --- | --- | --- | --- | --- | --- | --- | --- | --- |
| **STATUS** | 2/2 | 2/3 | 2/4 | 3/3 | 3/4 | 4/4 | 2* | 3* | 4* | HWE-P | Genotype | Allele |
| AD outbred (N=164) | --- | 6  (3.7%) | 1 (0.6%) | 59  (36.0%) | 80 (48.8%) | 18 (11.0%) | 7  (2.1%) | 204  (62.2%) | 117 (35.7%) | 0.842 | **0.008** | 0.315 |
| AD inbred (N=29) | --- | --- | 2 (6.9%) | 17 (58.6%) | 7 (24.1%) | 3  (10.3%) | 2  (3.4%) | 41  (70.7%) | 15  (25.9%) | 0.390 |  |  |
| AD Total (N=193) | --- | 6 (3.1%) | 3 (1.6%) | 76 (39.4%) | 87 (45.1%) | 21 (10.9%) | 9  (2.3%) | 245 (63.5%) | 132 (34.2%) | 0.997 |  |  |
| CTL outbred (N=159) | 1 (0.6%) | 19 (12.0%) | 3  (1.9%) | 106 (66.7%) | 27 (17.0%) | 3  (1.9%) | 24 (7.5%) | 258 (81.1%) | 36  (11.3%) | 0.998 | 0.765 | 0.506 |
| CTL inbred (N=24) | --- | 5 (20.8%) | 1 (4.2%) | 14 (58.3%) | 4 (16.7%) | --- | 6  (12.5%) | 37 (77.1%) | 5  (10.4%) | 1.000 |  |  |
| CTL Total (N=183) | 1 (0.5%) | 24 (13.1%) | 4 (2.2%) | 120 (65.6%) | 31 (16.9%) | 3 (1.6%) | 30 (8.2%) | 295 (80.6%) | 41 (11.2%) | 0.995 |  |  |
| TOTAL (N=376) | 1 (0.3%) | 30 (8.0%) | 7 (1.9%) | 196 (52.1%) | 118  (31.4%) | 24  (6.4%) | 39 (5.2%) | 540 (71.8%) | 173  (23.0%) |  |  |  |

E)

| **FIN** | ***APOE* genotype** | | | | | | ***APOE* allele** | | |  |  |  |
| --- | --- | --- | --- | --- | --- | --- | --- | --- | --- | --- | --- | --- |
| **STATUS** | 2/2 | 2/3 | 2/4 | 3/3 | 3/4 | 4/4 | 2* | 3* | 4* | HWE-P | Genotype | Allele |
| AD outbred (N=92) | --- | 11  (12.0%) | 1  (1.1%) | 30 (32.6%) | 37  (40.2%) | 13 (14.1%) | 12  (6.5%) | 108 (58.7%) | 64  (34.8%) | 0.569 | 0.214 | 0.475 |
| AD inbred (N=18) | --- | 1  (5.6%) | 1 (5.6%) | 10 (55.6%) | 4 (22.2%) | 2  (11.1%) | 2  (5.6%) | 25 (69.4%) | 9 (25.0%) | 0.908 |  |  |
| AD Total (N=110) | --- | 12 (10.9%) | 2 (1.8%) | 40 (36.4%) | 41 (37.3%) | 15 (13.6%) | 14 (6.4%) | 133 (60.5%) | 73 (33.2%) | 0.708 |  |  |
| CTL outbred (N=96) | --- | 6  (6.3%) | 3 (3.1%) | 44  (45.8%) | 36 (37.5%) | 7  (7.3%) | 9  (4.7%) | 130  (67.7%) | 53  (27.6%) | 0.996 | 0.057 | **0.012** |
| CTL inbred (N=13) | --- | 3 (23.1%) | --- | 5 (38.5%) | 2  (15.4%) | 3  (23.1%) | 3  (11.5%) | 15 (57.7%) | 8 (30.8%) | 0.554 |  |  |
| CTL Total (N=109) | --- | 9  (8.3%) | 3 (2.8%) | 49 (45.0%) | 38 (34.9%) | 10  (9.2%) | 12 (5.5%) | 145 (66.5%) | 61 (28.0%) | 0.994 |  |  |
| TOTAL (N=219) | --- | 21  (9.6%) | 5  (2.3%) | 89 (40.6%) | 79  (36.1%) | 25  (11.4%) | 26  (5.9%) | 278  (63.5%) | 134  (30.6%) |  |  |  |

F)

| **YRI** | ***APOE* genotype** | | | | | | ***APOE* allele** | | |  |  |  |
| --- | --- | --- | --- | --- | --- | --- | --- | --- | --- | --- | --- | --- |
| **STATUS** | 2/2 | 2/3 | 2/4 | 3/3 | 3/4 | 4/4 | 2* | 3* | 4* | HWE-P | Genotype | Allele |
| AD outbred (N=83) | 1 (1.2%) | 9  (10.8%) | 4 (4.8%) | 30 (36.1%) | 33 (39.8%) | 6  (7.2%) | 15 (9.0%) | 102 (61.5%) | 49 (29.5%) | 0.999 | N.D | N.D |
| AD inbred (N=1) | --- | 1 (100.0%) | --- | --- | --- | --- | 1 (50.0%) | 1 (50.0%) | --- | N.D. |  |  |
| AD Total (N=84) | 1 (1.2%) | 10 (11.9%) | 4 (4.8%) | 30 (35.7%) | 33 (39.3%) | 6  (7.1%) | 16 (9.5%) | 103 (61.3%) | 49 (29.2%) | 0.996 |  |  |
| CTL outbred (N=925) | 13 (1.4%) | 128 (13.8%) | 44 (4.8%) | 431 (46.6%) | 274 (29.6%) | 35 (3.8%) | 198 (10.7%) | 1264 (68.3%) | 388 (21.0%) | 0.961 | 0.207 | 0.553 |
| CTL inbred (N=11) | --- | 1  (9.1%) | --- | 6 (54.6%) | 2 (18.2%) | 2 (18.2%) | 1  (4.5%) | 15 (68.2%) | 6 (27.3%) | 0.843 |  |  |
| CTL Total (N=936) | 13 (1.4%) | 129 (13.8%) | 44 (4.7%) | 437 (46.7%) | 276 (29.5%) | 37 (4.0%) | 199 (10.6%) | 1279 (68.3%) | 394 (21.0%) | 0.983 |  |  |
| TOTAL (N=1020) | 14 (1.4%) | 139 (13.6%) | 48 (4.7%) | 467  (45.8%) | 309 (30.3%) | 43 (4.2%) | 215 (10.5%) | 1382 (67.8%) | 443  (21.7%) |  |  |  |

G)

| **ECD** | ***APOE* genotype** | | | | | | ***APOE* allele** | | |  |  |  |
| --- | --- | --- | --- | --- | --- | --- | --- | --- | --- | --- | --- | --- |
| **STATUS** | 2/2 | 2/3 | 2/4 | 3/3 | 3/4 | 4/4 | 2* | 3* | 4* | HWE-P | Genotype | Allele |
| AD outbred (N=220) | --- | 11 (5.0%) | 5 (2.3%) | 104 (47.3%) | 85 (38.6%) | 15 (6.8%) | 16 (3.6%) | 304 (69.1%) | 120  (27.3%) | 0.997 | 0.409 | 0.569 |
| AD inbred (N=117) | --- | 7  (6.0%) | 2 (1.7%) | 65 (55.6%) | 33 (28.2%) | 10 (8.6%) | 9  (3.8%) | 170 (72.6%) | 55  (23.5%) | 0.770 |  |  |
| AD Total (N=337) | --- | 18 (5.3%) | 7 (2.1%) | 169 (50.1%) | 118 (35.0%) | 25 (7.4%) | 25 (3.7%) | 474 (70.3%) | 175 (26.0%) | 0.991 |  |  |
| CTL outbred (N=238) | 2 (0.8%) | 25 (10.5%) | 2 (0.8%) | 166 (69.8%) | 39 (16.4%) | 4  (1.7%) | 31 (6.5%) | 396 (83.2%) | 49  (10.3%) | 0.980 | 0.333 | 0.596 |
| CTL inbred (N=96) | --- | 11 (11.5%) | 1 (1.0%) | 60 (62.5%) | 24 (25.0%) | --- | 12 (6.3%) | 155 (80.7%) | 25  (13.0%) | 0.590 |  |  |
| CTL Total (N=334) | 2 (0.6%) | 36 (10.8%) | 3 (0.9%) | 226 (67.7%) | 63 (18.9%) | 4 (1.2%) | 43 (6.4%) | 551 (82.5%) | 74 (11.1%) | 0.972 |  |  |
| TOTAL (N=671) | 2 (0.3%) | 54 (8.1%) | 10 (1.5%) | 395 (58.9%) | 181 (27.0%) | 29 (4.3%) | 68 (5.1%) | 1025 (76.4%) | 249  (18.5%) |  |  |  |

H)

| **ACD** | ***APOE* genotype** | | | | | | ***APOE* allele** | | |  |  |  |
| --- | --- | --- | --- | --- | --- | --- | --- | --- | --- | --- | --- | --- |
| **STATUS** | 2/2 | 2/3 | 2/4 | 3/3 | 3/4 | 4/4 | 2* | 3* | 4* | HWE-P | Genotype | Allele |
| AD outbred (N=123) | 2 (1.6%) | 15 (12.2%) | 5 (4.1%) | 39 (31.7%) | 52 (42.3%) | 10 (8.1%) | 24 (9.8%) | 145 (58.9%) | 77 (31.3%) | 0.918 | 0.947 | 0.783 |
| AD inbred (N=47) | 1 (2.1%) | 4  (8.5%) | 1 (2.1%) | 16 (34.0%) | 22 (46.8%) | 3 (6.4%) | 7 (7.4%) | 58 (61.7%) | 29 (30.9%) | 0.801 |  |  |
| AD Total (N=170) | 3 (1.8%) | 19 (11.2%) | 6 (3.5%) | 55 (32.4%) | 74 (43.5%) | 13 (7.6%) | 31 (9.1%) | 203 (59.7%) | 106 (31.2%) | 0.585 |  |  |
| CTL outbred (N=181) | 2 (1.1%) | 20 (11.1%) | 11 (6.1%) | 111 (61.3%) | 35 (19.3%) | 2 (1.1%) | 35 (9.7%) | 277 (76.5%) | 50 (13.8%) | 0.516 | 0.134 | 0.569 |
| CTL inbred (N=47) | --- | 8 (17.0%) | --- | 23 (48.9%) | 15 (31.9%) | 1 (2.1%) | 8 (8.5%) | 69 (73.4%) | 17 (18.1%) | 0.641 |  |  |
| CTL Total (N=228) | 2 (0.9%) | 28 (12.3%) | 11 (4.8%) | 134 (58.8%) | 50 (21.9%) | 3 (1.3%) | 42 (9.2%) | 346 (75.9%) | 67 (14.7%) | 0.790 |  |  |
| TOTAL (N=398) | 5 (1.3%) | 47 (11.8%) | 17 (4.3%) | 189 (47.5%) | 124 (31.2%) | 16 (4.0%) | 74 (9.3%) | 549 (69.0%) | 173 (21.7%) |  |  |  |

**Table S15. Private minor allele homozygote variants in ADSP consanguineous subjects.** The table reports the minor allele homozygote variants identified in ADSP/GWAS-matched consanguineous subjects that were not found in homozygote state in the ADSP subjects used for the validation phase.

**Supplementary Figure 1. Pre-imputation allele-frequency correlation in Europeans (North-Western, NWE; Ashkenazi Jewish, AJE; South-Eastern, SEE), Africans (AFR) and African-Caribbeans (AFR-CAR).** The plots show the allele frequency correlation between QCed GWAS group and Haplotype Reference Consortium (HRC) Panel r1.1 (excluding chromosome X) for European groups. African population from 1000 Genomes Phase 3 project for African and CAAPA panel for African-Caribbeans. Correlation values (r2) range from 0.936 (AJE-Group 4) to 0.997 (NWE-Group 1).


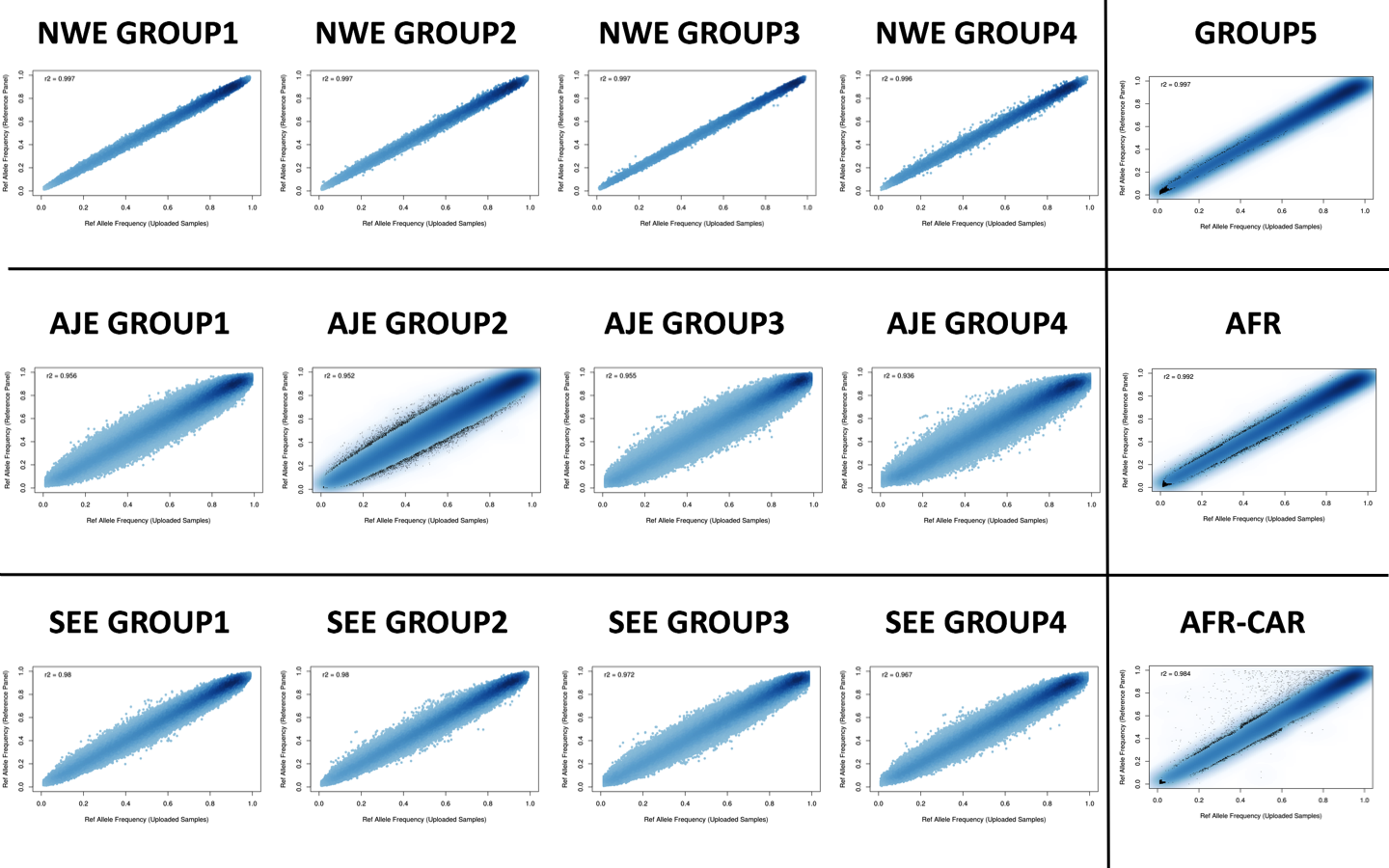

Supplement: Supplementary file 1 [file Data_Sheet_1.docx]
